# Mapping Allosteric Communication in the Nucleosome with Conditional Activity

**DOI:** 10.1021/acs.jcim.6c00317

**Published:** 2026-02-24

**Authors:** Augustine C. Onyema, Chukwuebuka Dikeocha, Rutika Patel, Jonathan Moussa, Sharon M. Loverde

**Affiliations:** † Department of Chemistry, College of Staten Island, 2009 The City University of New York, 2800 Victory Boulevard, Staten Island, New York 10314, United States; ‡ Department of Computer Science, College of Staten Island, The City University of New York, 2800 Victory Boulevard, Staten Island, New York 10314, United States; § 1757 The Molecular Science Software Institute (MolSSI), Blacksburg, Virginia 24060, United States; ∥ Ph.D. Program in Chemistry, Biochemistry, and Physics, The Graduate Center of the City University of New York, New York, New York 10016, United States; ⊥ Ph.D. Program in Biochemistry, The Graduate Center of the City University of New York, New York, New York 10016, United States

## Abstract

The nucleosome core particle (NCP) regulates genome accessibility through dynamic allosteric communication between histone proteins and DNA. Building on the concept of conditional activity introduced by Lin (2016), we use molecular dynamics simulations and develop an open-source Python library, CONDACT (CONDitional ACTivity), to quantify time-resolved kinetic correlations in nucleosome systems. We analyze long-time simulations of the nucleosome core particle, including two different DNA sequences, the Widom-601 (PDB ID: 3LZ0) and ASP (alpha-satellite palindromic) sequences (PDB ID: 1KX5). By tracking dihedral angle transitions, we identify residues with high dynamical memory and map inter-residue communication pathways across histone subunits and DNA. Our analysis reveals kinetically connected domains involving post-translational modification sites, oncogenic mutation sites, and DNA contact regions, with dynamic coupling observed over distances up to 7.5 nm. These findings offer new insight into the long-range allosteric behavior of the nucleosome and its potential role in regulating chromatin accessibility. Quantifying this allosteric behavior potentially identifies targetable residues and domains for therapeutic intervention.

## Graphical abstract

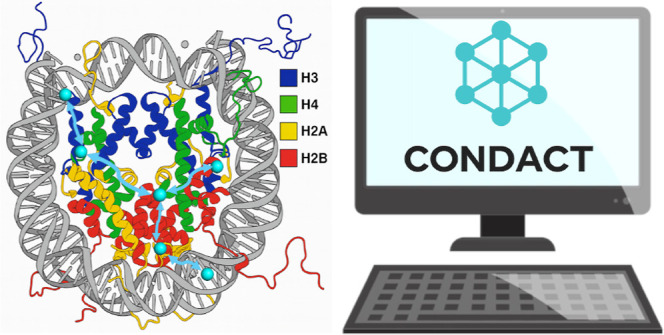

## 1 Statement of Significance

The nucleosome regulates DNA accessibility and plays a key role in epigenetic control of gene expression; yet, the kinetic mechanism by which local modifications influence distant regions remains poorly understood. Here, we identify kinetically coupled domains that include post-translational modification and oncogenic mutation sites. We uncover networks of allosteric signaling pathways that provide fundamental insight into how the nucleosome communicates.

## 2 Introduction

The shape of biomolecules, such as proteins and nucleic acids, is intimately linked to their function. Proteins often undergo conformational changes upon binding nucleic acids to perform tasks including catalysis,^1,2^ signaling,^3^ and transport.^4^ Similarly, nucleic acids dynamically reorganize to modulate DNA accessibility^5^ or RNA structure during translation.^6^ These conformational shifts are frequently triggered by specific ligands or environmental cues, allowing molecules to function as biochemical sensors.^7^ For instance, enzymes may shift between the active and inactive states in response to cellular energy levels. At the same time, transcription factors can bind to double-stranded DNA in the nucleosome, altering the gene expression patterns. Traditionally, allostery has been associated with structural transitions in response to external stimuli; however, the concept of dynamic allostery has expanded this view by focusing on changes in flexibility and disorder within the same overall conformational space.^8^ Such subtle dynamic modulations—such as fluctuations in dihedral angles or the transient breaking of hydrogen bonds—can propagate signals across a molecule without necessitating large-scale structural rearrangements, thus providing a mechanistic basis for kinetically correlated residue pairs consistent with long-range communication.^9^

A range of computational and theoretical methods have been developed to characterize correlated structural changes in biomolecules, spanning from elastic network models for proteins^10^ to base-pair-level descriptors of DNA deformability.^11^ Additional approaches include principal component analysis (PCA), which captures dominant modes of motion in biomolecules,^12^ dynamical network models^13^ that map residue communication pathways and capture local coupling dynamics, including for DNA base pair orientations,^14^ and graph-theoretical frameworks that represent molecular structures as networks to identify key topological features underpinning allostery and conformational dynamics.^15^ Time-lagged independent component analysis (tICA) identifies slow collective motions and potential allosteric pathways by capturing time-correlated features in molecular dynamics trajectories.^16^ tICA can serve as a basis for the development of Markov State Models (MSMs) that capture long-time dynamics; for example, our laboratory has applied MSMs to map the long-time scale conformational dynamics of histone tails, revealing metastable states and transition pathways that may be subtly altered with post-translational modifications (PTMs).^17^ MSMs have also been used in combination with coarse-grained models to characterize the dynamic landscape of nucleosome assembly.^18^ Additionally, methods in machine learning, such as diffusion maps, have been employed to characterize the translocational and rotational motion of complex polymers, including DNA within the nucleosome.^19^

To track changes in the molecular degrees of freedom, concepts like the mutual information theory have been used, which measures the amount of information transmission between two residues in a system, regardless of the functional form of their relationship.^20^ While heterogeneity in a structure can be addressed using parameters such as normalized mutual information,^21^ heterogeneities in dynamics can be further understood by adapting methods from glassy systems.^22,23^ By combining mutual-information measures of structural covariation with glassy-system tools that capture transient, locally heterogeneous motions, we get a unified framework to map how allosteric signals are transmitted. Together, these approaches help bridge structural and dynamic heterogeneities by identifying both static correlations and temporally coordinated motions across biomolecular systems. Graph models, in which specific atoms in amino acids and nucleotides are depicted as nodes and their connections as edges, have also been used to trace the route between sites of allosteric induction and the site of the downstream effector.^2,24,25^ The resulting node and edge connection generates a weighted graph system whose substructures of highly correlated residues can be determined with the Girvan–Newman algorithm.^26^ The shortest path (Floyd–Warshall algorithm) linking allosteric sites aids the understanding of information transmission in the constructed network.^25,27^ Indeed, regions of proteins or DNA may behave in a more correlated or more liquid-like way when incorporating key features that are sometimes ignored in the approaches mentioned above.

Here, we focus on side-chain dihedral angles, a feature not commonly incorporated in previously discussed approaches. However, the timing transitions of changes in the side-chain dihedral angle can be used to characterize how distant regions in proteins communicate, as was shown with correlation of all rotameric and dynamical states (CARDS).^21^ We next extend a similar approach to characterize the interface between protein–DNA, incorporating the base-sugar torsion angle of the DNA, which is known to be coupled to distinct transitions in the DNA structure.^28,29^ Various biophysical techniques, including mass spectroscopy, nuclear magnetic resonance (NMR), and other computational techniques like molecular dynamics simulations, have been employed to study the allosteric properties of proteins and nucleic acids.^30–34^ Flexible and interacting sites in proteins have been seen using highly sensitive hydrogen–deuterium exchange mass spectroscopy.^35^ These techniques capture the dynamics of different domains of the molecule by examining dynamically correlated regions by measuring a specific degree of freedom.

The nucleosome core particle (NCP) is one such allosteric complex whose dynamics are affected by allosteric regulators, post-translational modifications, and interactions with transcription and remodelers. Molecular dynamics simulations revealed that histone variants macroH2A and an L1-loop mutant enhance nucleosome stability by strengthening dimer–dimer and histone–DNA interactions, reducing DNA breathing, and reinforcing compact conformations.^36^ In contrast, H2A.Z introduces slight energetic changes that reconfigure allosteric communication networks, potentially modulating chromatin accessibility and the regulatory response. Our laboratory has observed that acetylation of the H2B tail alters tail interaction with the double-stranded DNA in the NCP.^37^ Post-translational modification at specific amino acid residues has altered NCP breathing motions, acidic patch binding, or promoted hexasome formation.^38–41^ Single-pair FRET (spFRET) has been used to show that H3K56ac significantly enhances nucleosomal DNA breathing through a 2-fold unwrapping of the first 20 base pairs.^38^ Zhang and colleagues saw that H4K16 acetylation disrupts the H4 tail’s anchoring interaction with the neighboring nucleosome acidic patch, weakening internucleosome contacts,^39^ while Luo et al. explained that monoubiquitination of H2BK120 weakens intrinsic nucleosome stability to promote FACT recruitment that facilitates transcription.^42^ Besides PTMs, mutations in the histone core, including those implicated in certain cancers, have been shown to alter the hydrogen-bond dynamics in the core histones, indicating that information is transmitted within the NCP.^43^ Critical disruptions at nucleosome interaction interfaces have been seen to rewire chromatin regulation in histone-associated oncogenic systems (H2BE76K, H3K27M, H3K36M, and H3G34R), contributing to oncogenesis.^44^ Nucleosome interaction histone–histone, histone–DNA, and histone–protein interfaces were rewired, affecting dynamics and changing the nucleosome allostery. Several additional PTMs and mutations highlighted in our analysis occur at functionally critical residues identified through conditional activity mapping. For instance, H3R42—a methylation site within the DNA entry/exit latch region—exhibits high inter-residue conditional activity with other PTM sites such as H3K27 and H3K36, suggesting coordinated dynamics across the nucleosome. H2BE76, a recurrent oncogenic mutation site, shows strong coupling with H3R131 and H2AH31, indicating long-range communication between the histone cores. These residues, found at the histone–DNA interface, in the acidic patch, or at the tetramer–dimer junction, form kinetically linked networks that are sensitive to both PTMs and mutation-induced perturbations, with potential implications for nucleosome stability and chromatin regulation.

Given the extensive network of allosterically linked PTM and mutation sites, it is critical to adopt a framework that captures the underlying kinetic relationships driving these long-range correlations. Geometric properties, such as distances and dihedral angles, derived from molecular dynamics simulation trajectories, were utilized by Vögele and co-workers in their software package, Python ENSemble Analysis (PENSA), to comprehensively investigate changes in biomolecular conformational ensembles.^45^ Bheemireddy and colleagues proposed ComPASS (Communication Pathway AnalysiS within macromolecular Systems), a network-based framework that uses molecular dynamics simulations to map information transmission in biomolecular complexes.^46^ They integrated multiple residue-level features, including dynamical correlations, noncovalent interaction persistence, distance fluctuations, and spatial proximity, into a single weighted communication network. By representing residues as nodes and edges as coupling strengths, they identified the most efficient communication routes in the system. Lin et al. proposed the use of kinetic properties in their time-related kinetic correlation, which they termed conditional activity, to trace the correlation between domains in the catabolite activator protein (CAP).^9^ This uniquely reveals a hidden allosteric link in CAP, a directional, dynamic coupling from the cAMP-binding site of one monomer to the DNA-binding site of the other, undetectable by mutual information or RMSD-based structural analysis. In a sister study, Manley and Lin revealed that Ras isoforms use distinct combinations of kinetic (timing-based) and thermodynamic (entropy-based) allosteric pathways centered on switches I and II to regulate effector interactions and catalytic activity, explaining isoform-specific functional differences.^47^ To identify residues whose changes in the degree of freedom are significant in the overall dynamics of the NCP and reveal kinetically correlated domains in the system, we introduce the open-source Python library CONDACT, based on the concept of conditional activity proposed by Lin et al.^9^ CONDACT, coined from CONDitional ACTivity, traces time-related changes of properties in molecular dynamics simulation trajectories that extensively sample changes of different geometric states to deduce kinetically significant residues and domains and trace the route of information transmission between the site of allosteric induction and the site of the effector response. The library is sensitive to detecting kinetic correlations in pairwise residues over nanometer-scale distances.

## 3 Methods

### 3.1 Conditional Activity

Molecular dynamics is characterized by changes in various degrees of freedom, which include dihedral angles, distances, and electrostatic contacts, among others. While using molecular dynamics simulation to sample the potential energy surface of biomolecular systems, different residues and domains adopt different states at any given time during the system’s dynamics. The dynamic relationship among different parts of heterogeneous systems, such as proteins, has been computed using different correlation tools. Mutual information (MI) theory has been widely used to understand the correlation between residues *X* and *Y* in protein systems. The MI of an observable degree of freedom of *X* and *Y* is defined as the sum of the entropies of *X* and *Y* minus the combined entropy of the complexes *X* and *Y*,^48,49^ as follows: $MI\left(X,Y\right)=\sum_{x\epsilon X}P\left(X\right)\ln\left[P\left(X\right)\right]+\sum_{y\epsilon Y}P\left(Y\right)\ln\left[P\left(Y\right)\right]-\sum_{x\epsilon X}\sum_{y\epsilon Y}P\left(X,Y\right)\ln\left[P\left(X,Y\right)\right]$ . Here, *P*(*X*), *P*(*Y*), and *P*(*X*,*Y*) are the probabilities of *X*, *Y,* and *X*,*Y,* respectively. The MI, therefore, gives the amount of thermodynamic or entropic information that *X* shares with *Y*.

However, some residues or domains in some biological systems exhibit large-scale kinetic correlation with or without noticeable physical changes in their entropic or thermodynamic properties. The correlation of these residues can also be evaluated using kinetic information, termed conditional activity.^9,47^ Degrees of freedom like distance, angle, electrostatic contacts, and backbone or side-chain dihedral angle change states, and the changes in these states serve as a kinetic clock to track kinetically associated residues/domains. These degrees of freedom are usually characterized as being in one finite state at any point in time during a molecular dynamics simulation. The time at which these degrees of freedom of a residue, *X*, change from one finite state to another is termed the transition time of that residue (*T*_*x*_). If two residues *X* and *Y* have a degree of freedom that changes among different finite states, the *i*th transition time of *X* or *Y* during a simulation denoted as *T*(*X*, *i*) and *T*(*Y*, *i*), respectively, is the time at which the degree of freedom of *X* or *Y* changes from one finite state to another finite state. The waiting time of the residue *X*, denoted as *W*(*X*, *t*), is the time interval between the time of the *i*th transition and the (*i* + 1)^th^ transition of X as follows: *W*(*X*, *t*) = *T*(*X*, *i* + 1) – *T*(*X*, *i*). The transition and waiting times are independent of the state of the degree of freedom of interest but depend on the time of transition of that degree of freedom. The conditional activity is related to the persistence time of specific residues and the exchange time between the two residues.

The mean persistence time is the average time a degree of freedom of a residue stays in a specific finite state during a simulation. It is therefore the average waiting time starting from a random time *t* until the next transition of the degree of freedom of *X*. To accurately compute the persistence time of a residue, the number of transitions by the degree of freedom must be far greater than 1 (*N* ≫1). Therefore, the conditional activity between residues is best determined for a well-sampled trajectory with an observation time τ≡*T*(*X*, *N*(*X*)) in which there are multiple transitions of the degree of freedom. The mean persistence time of *X*, τ_p_[*X*], is directly proportional to the mean-squared waiting times of *X* calculated from the corresponding list of transition times as follows: $\tau_{p}\left[X\right]=\frac{1}{2\tau }\sum_{i=1}^{N\left(X\right)}W({X,\;T\left(X,i\right)}^{2}$ . The mean-squared waiting time is the product of that waiting time and the probability of selecting that waiting time. The probability of selecting a specific waiting time is proportional to the same waiting time. The mean exchange time of the degree of freedom of the residue *X* following a transition of another residue *Y* (τ_p_[*X*]­[*Y*]) is the waiting time for *X* to undergo a transition after an (*i* + 1)^th^ transition in *Y*. The exchange time of *X* after a transition in *Y* is proportional to the probability of the waiting time between the *i*th and (*i* + 1)^th^ transitions of *Y* defined as $\tau_{p}\left[X\right]\left[Y\right]=\frac{1}{\tau }\sum_{i=1}^{N\left(Y\right)-1}W\left(X,T\left(Y,i+1\right)\right)W\left(Y,T\left(Y,i\right)\right)$ . The conditional activity of X with respect to *Y* (*A*[*X*]­[*Y*])­is the negative logarithm of the ratio of the exchange time of *X* after a transition in *Y* (τ_p_[*X*]­[*Y*]) to the persistence time of *X* (τ_p_[*X*]) as shown below, $A\left[X\right]\left[Y\right]\equiv -\ln\left[\frac{\tau_{x}\left[X\right]\left[Y\right]}{\tau_{p}\left[X\right]}\right]$ .

Depending on the systems of interest, conditional activity values can be positive or negative. If the degree of freedom of a residue *X* is independent of another residue *Y*, then the conditional activity of *X* on *Y* is equal to zero (*A*[*X*]­[*Y*] = 0). The more positive the conditional activity (*A*[*X*]­[*Y*] > 0), the higher the probability that a transition in *Y* leads to a corresponding transition in *X*. However, if *A*[*X*]­[*Y*] < 0, a transition in *X* is less likely to promote a corresponding transition in *Y*. Furthermore, the conditional activities of the degree of freedom of two residues are not commutative, i.e., *A*[*X*]­[*Y*] ≠ *A*[*Y*]­[*X*]. The diagonal of the conditional activity matrix is the conditional activity of a residue on itself (*A*[*X*]­[*X*]), and this represents the dynamic memory of that residue. If *A*[*X*]­[*X*] ≈ 0, then the successive waiting times of *X* are independent of the history of *X*; hence, the transitions of *X* are random following a Poisson distribution. The dynamical memory of a specific residue *X* is not interaction-specific and therefore does not deduce the direction of interaction between *X* and another residue *Y* (*X* to *Y*). The dynamical memory only identifies residues whose side-chain dynamics are statistically significant. The direction of interaction between a residue *X* and another residue *Y* (*A*[*X*]­[*Y*]) can be obtained from the off-diagonal entries of the conditional activity matrix, in which the more positive *A*[*X*]­[*Y*], the higher the probability that the side-chain dynamics of *Y* influence the side-chain dynamics of *X*. The kinetically connected domain can also be traced from the conditional activity matrix. The principal eigenvector of the symmetrized conditional activity matrix predicts the system’s dynamically connected domains. This shows domains with the largest modes of correlated fluctuations, identifying residues that make the largest contributions to these fluctuations.

### 3.2 State Assignment to Degree of Freedom

We apply our conditional activity module to six systems: two enzymes and four nucleosome core particle (NCP) systems. Lysozyme (PDB ID: 1AKI)^50^ and the third domain of synaptic protein PSD-95 (PDZ3 with PDB ID: 1BFE)^51^ are the enzyme systems (Table S1a), while the nucleosome core particles (NCPs) contain the alpha satellite sequence ASP (PDB ID: 1KX5),^52^ the Widom-601 sequence (PDB ID: 3LZ0),^53^ and two mutants: H4R92T and H2BE76K of the 1KX5 system (Table S1b). The enzyme systems were selected because their mechanisms have been extensively studied, including the residues in their active sites (binding groups and catalytic groups) and allosteric sites.^54^ Also, the enzyme results could be used to compare the work of other authors who performed kinetic analyses of the same protein.^9^ The lysozyme and PSD-95 systems are analyzed to validate our CONDACT code and compare with the previous results reported in Lin et al.^9^ The foundational 2.8 Å X-ray structure of the nucleosome core particle established the histone octamer architecture and DNA path, providing a structural baseline for mechanistic studies of dynamics.^55^ The H4R92T and H2BE76K mutations were selected because these highly probable oncogenic histone mutations have been seen to destabilize the core of the NCP.^43^ Details of the system parametrization, simulation parameters, and replica information can be found in the Supporting Information. The enzyme systems were each run for up to 3 μs, while each NCP system was run for 6 μs.

To understand the conditional activity of both proteins and DNA in the nucleosome core particle (NCP), we utilized dihedral angles as a measure of the degree of freedom. The large size of the NCP encourages slow dynamics of the systems, preserving the secondary structure of the core histones and double-stranded DNA. To this end, side-chain dihedral angles (χ_1_) are used as the degree of freedom for the proteins (Figure 1a). In contrast, the base-sugar nucleoside dihedral angles (O4^I^–C1–N9–C4 for purine and O4^I^–C1–N1–C2 for pyrimidine) are used as the degree of freedom for the double-stranded DNA (Figure 1b,c). The side chains of amino acids and the base-sugar backbone of the nucleoside can adopt different states driven by short-range or long-range interactions of the residues. In essence, amino acids glycine and alanine, devoid of a side-chain dihedral angle (χ_1_), are ignored in this study. The secondary angles of all negative dihedral angles are utilized to make the 0–360° range. The probability density of the dihedral angles of all amino acids or DNA in the enzymes/NCP system across all simulations was calculated (Figure 1d,e), and states were assigned to each peak. The protein dihedral angles are assigned three states (*X*, *Y*, and *Z*), while the DNA base-sugar nucleoside dihedral angles are assigned two states (*S* and *A* for syn and anti conformations, respectively) as shown in Figure 1b,c. For amino acids, 0°≤ θ < 120° was assigned state *X*, 120°≤ θ < 240° was assigned state *Y*, and 240°≤ θ < 360° was assigned state *Z*. Similarly, for the nucleotides in the double-stranded DNA, 0°≤ θ < 140° and 330°≤ θ < 360° were assigned state *S* and 140°≤ θ < 330° was assigned state *A*. To ensure robust analysis, only residues with ten or more transitions are included in the conditional activity analysis because different states of the degree of freedom have been efficiently sampled.

**Figure 1:**
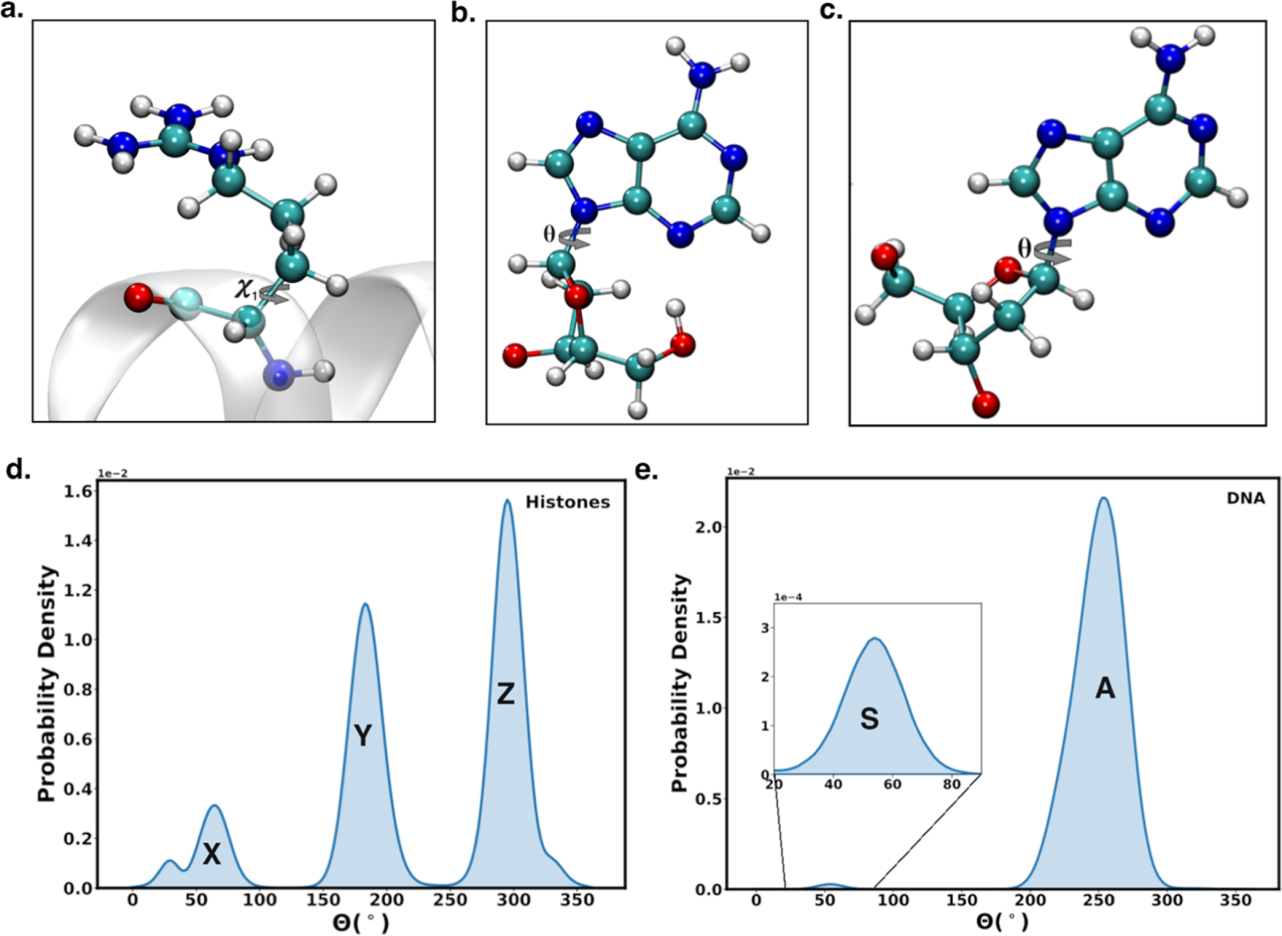
Dynamics of the dihedral angle of amino acid side chain in protein and base-sugar dihedral angle in DNA is used as degree of freedom: (a) first side-chain dihedral angle for proteins (χ_1_). The base-sugar dihedral angle (θ) for DNA in the (b) syn conformation and the (c) anti conformation. (d) Probability density distribution of the side-chain dihedral angles for all amino acids in the system for all simulations. The probability density shows three distinct peaks representing three states *X*, *Y*, and *Z*. (e) Probability density distribution of the base-sugar dihedral angles for all nucleotides in the double-stranded DNA for all simulated systems. The DNA probability density distribution of the dihedral angle reveals two defined peaks, which represent the syn (*S*) state and the anti (*A*) state.

## 4 Results

### 4.1 Conditional Activity Reveals Residues with High Dynamical Memory

The conditional activity of all systems reveals amino acids or nucleotide residues in the enzyme and NCP systems, which are characterized by high dynamical memory. The dynamics of residues with high dynamical memory do not follow a Poisson distribution; hence, their transitions are not random or successive transitions depend on previous dynamics. The convergence of the conditional activity matrices was assessed in detail in the Supporting Information. For the enzyme systems, the residues with the highest dynamical memory are in the active site for lysozyme and in both the active and allosteric sites for PDZ3 (Figure S1a,b and Text S1.3), consistent with previously reported results by Lin et al.^9^ For the NCPs, the dynamical memory for the degree of freedom is heterogeneously distributed throughout the systems (Figure 2a,b), revealing statistically significant dynamics of amino acid side chains in the histone and base-sugar dihedral angle of the double-stranded DNA. These residues are in different domains on the histones, including the alpha-1 (α1) helix, alpha-2 (α2), and Loop1 (L1) of histone H4, the H3-αN helix, the acidic patch, and the SHL-2 and SHL-4 regions in both the 1KX5 and/or the 3LZ0 systems. Single-molecule force spectroscopy revealed that nucleosomes unwrap asymmetrically from entry/exit sites and progress through SHL regions under tension, contextualizing the elevated dynamical memory observed at SHL-2 and SHL-4.^56^ It is worth noting that all histone subunits in the NCP contained residues with high dynamical memory at the core, tail, histone–histone interface, and histone–DNA interface. Table 1 shows the percentage of residues with a dynamical memory of at least 50% relative to the residue with the highest dynamical memory, which was approximately 8.0 across all systems.

**Figure 2:**
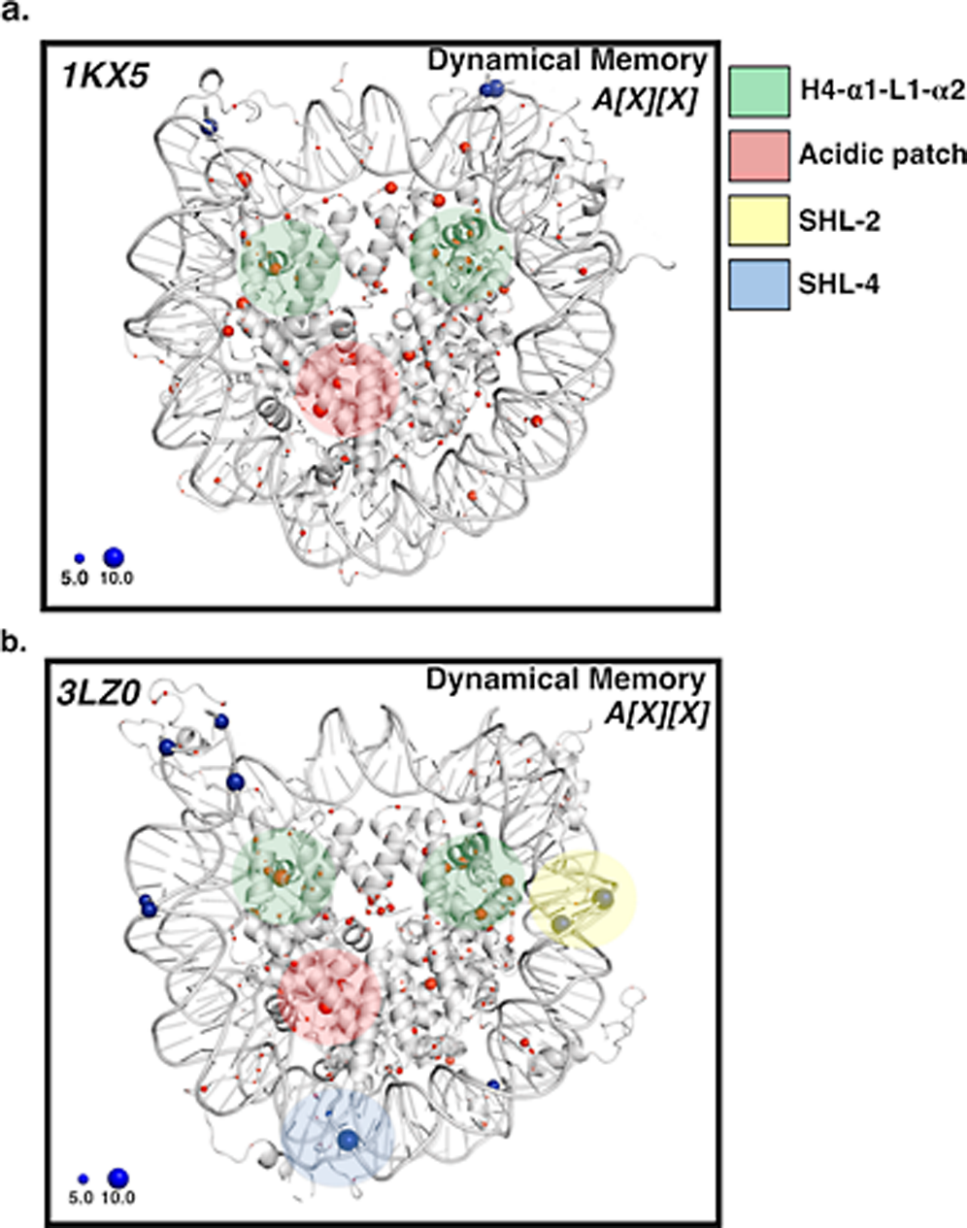
Dynamical memory (A­[X]­[X]) of charged residues in the NCP for (a) alpha satellite sequence (1KX5) and (b) Widom-601 sequence (3LZ0). The sphere sizes are proportional to the dynamical memory. Residues with the higher dynamical memory at the H4-α1, H4-α2, and H4-L1 domains are in the green-shaded region, the acidic patch residues are in the red-shaded region, the DNA SHL-2 region is in yellow, while the SHL-4 region is in blue. The residues in these regions modulate the binding of nucleosome binding chaperones or remodelers, and their mutations have been seen in some cancer types, like breast cancer and colorectal cancer.

**Table 1:** Percentage of Residues with Dynamical Memory Greater Than 4 in NCP^a^

	NCP tails	NCP core	H3	H4	H2A	H2B
1KX5	3.50	16.90	12.67	16.03	18.81	11.27
3LZ0	1.52	18.69	12.27	15.72	19.57	9.80

a Values reported in %.

In the 1KX5 system, residues on both copies of the H4-α1, H4-α2, H4-L1, and acidic patch show a very high dynamical memory (Figure 2a). The residues H4D24, H4R35, H4R36, and H4R40 on H4-α1 and H4K44 on H4-L1 show a high dynamical memory. Also, H4E53 and H4R55 on the α2 helix of H4.1 and H3R42 on the αN helix show statistically significant dynamics of their side-chain dihedrals. In the same light, H2AE56, H2AE64, and H2BD68, located in the acidic patch, have high dynamical memory.

The 3LZO system also has some dynamically active residues identical to those of the 1KX5 system. Both copies of histone H4 showed dynamically active residues on their α1 (H4R36 and H4R40) and α2 (H4E53 and H4R55) helices. Also, H3R63 on the α1-helix of both copies of histone H3 was dynamically active. The conditional activity of acidic patch residues in the 3LZ0 systems reveals that the transitions of H2AE56, H2AE64, H2AD91, H2BD68, and H2BE113 are not memoryless, as the dynamic memory was high. However, unlike the 1KX5 system, some nucleotides in the double-stranded DNA in the 3LZ0 systems, which are distant from the DNA terminals, show statistically significantly high dynamical memory. For example, guanine 54 (5^I^-3^I^ strand) and guanine 93 (3^I^-5^I^ strand) in the SHL-2 region and guanine 32 (5^I^-3^I^ strand) in the SHL-4 region showed high dynamical memory, which might indicate that the residues are slightly stressed when flipping between the syn and anti states.

The residues on helix H4-α1 have been implicated to interact with nucleosome remodelers and histone binding proteins. H4D24 has been seen to modulate the binding of chromatin remodelers like Chd1, disrupting the methylation of H4R20 by Suv4-20 homologue 1 (Set8/Suv4-20h1).^57^ Cryo-EM structures of Chd1 bound to nucleosomes show how remodelers engage the H4 tail/acidic-patch surfaces to stabilize sliding intermediates, aligning with the kinetically active H4/acidic-patch residues detected.^58^ The histone chaperone retinoblastoma-associated protein RbAp46 interacts with H4-α1 helix, with H4R35, H4R36, and H4R40 actively involved in its binding,^59^ while H4K44 has been seen to facilitate chromatin accessibility.^60^ Residues in the acidic patch of the nucleosome not only regulate binding of the nucleosome to some chaperones like FACT (in yeast), but their mutations have been implicated in different cancer types like colorectal cancer, head and neck cancer, and breast cancer.^61,62^ The SHL-2 and SHL-4 regions are the sites where histone H4 N-terminal tail interacts with the DNA, or the sites at which pioneer transcription factors like Sox2 bind with the double-stranded DNA.^63–65^

To deduce the direction of association between residues *X* and *Y*, the off-diagonal entries of the conditional activity matrix are used. The nucleosome is a highly allosteric complex with large-scale communication among residues in the histone and DNA. These pairs of communication are not only found in the histone core but also at the histone–DNA interface (Figures 3a,b and S2c,d). The top 1% of amino acids with the highest statistically significant inter-residue conditional activities include sites of oncogenic mutations and sites of post-translational modifications (Tables 2 and S2). Residues like H3R42, H3R131, H4R92, and H2BE76 were seen to have high inter-residue conditional activities affecting the side-chain dynamics of multiple residues on both the identical and other histone subunits.

**Figure 3:**
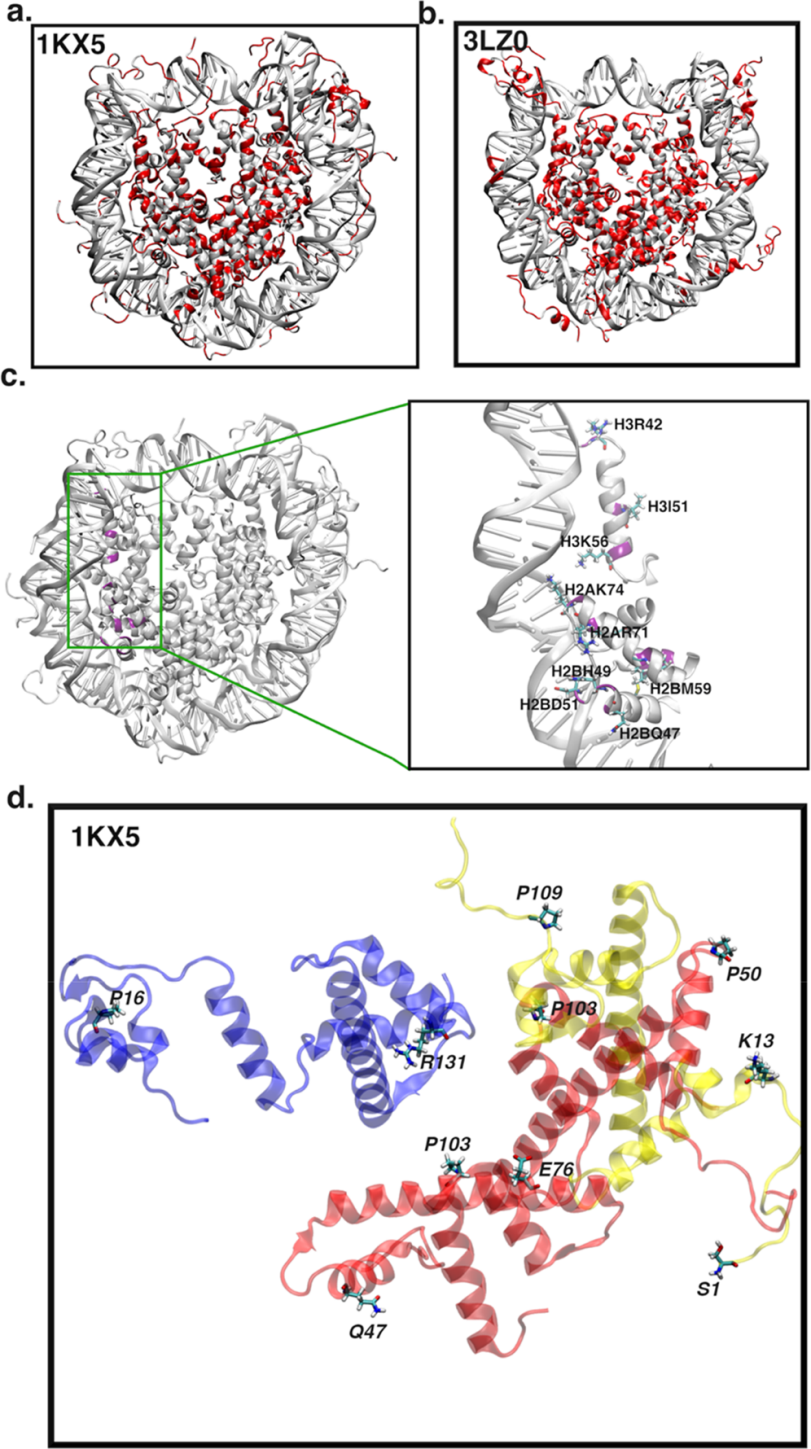
Inter-residue conditional activity (*A*[*X*]­[*Y*]) between residues in the NCP for (a) 1KX5 and (b) 3LZ0 systems. It shows all histone and DNA residues (in red) participating in highly correlated dynamic communication in the NCP, describing the NCP as a giant allosteric complex. (c) NCP showing H3R42 communication with other amino acids at the histone–DNA interface. This communication may affect DNA unwrapping or breathing motion. The positions of communication amino acid residues are in purple. (d) Positive inter-residue conditional activity between H3-R131 and other amino acids (*A*[*R*131]­[*Y*]) in the same or other histone subunits. Histone H3 in blue, H2A in yellow, and H2B in red. Side-chain dynamics show long-range correlations between H3-R131 and other amino acids, including H2B-E76 and H2A-K13.

**Table 2:** Statistically Significant Inter-Residue Conditional Activity of Residues at Oncogenic or Post-Translational Modification Sites for the 1KX5 System^a^ ^,^ ^b^ ^,^ ^c^ ^,^ ^d^ ^,^ ^e^

residue_*X*	mut_*X*	PTM_*X*	residue_*Y*	mut_*Y*	PTM_*Y*	*A*[*X*][*Y*]	p_value
H3-E105	yes	no	H2A-I102	no	no	3.042	0.0035
H2B-E113	yes	no	H3-V71	no	no	2.381	0.0020
H2B-E113	yes	no	H4-T71	no	no	2.613	0.0030
H3-K36	yes	yes	H2A-I102	no	no	3.316	0.0015
H3-L65	no	no	H3-R42	no	yes	5.393	0.0020
H3-T3	no	no	H2B-F70	yes	no	2.512	0.0010
H3-S10	no	no	H2B-F70	yes	no	2.350	0.0040
H4-R17	yes	no	H2A-H31	yes	no	2.639	0.0035
H2A-E92	yes	no	H2A-H31	yes	no	2.277	0.0055
H2A-L83	no	no	H2A-H31	yes	no	2.386	0.0010
H2B-H49	no	no	H2A-H31	yes	no	2.540	0.0035
H2B-L102	no	no	H2A-H31	yes	no	2.860	0.0025

a Mut-*X* is the site of oncogenic mutations of *X*.

b PTM-*X* is the site of post-translational modification of *X*.

c Mut-*Y* is the site of oncogenic mutations of *Y*.

d PTM-*Y* is the site of post-translational modification of *Y*.

e *A*[*X*]­[*Y*] is the conditional activity of *X* on *Y*.

Looking closely at the kinetic communication route for H3R42, CONDACT revealed communication with H3I51, H3K56, H2AK74, H2AR71, H2BH49, H2BD51, H2BQ47, and H2BM59, all of which are at the histone–DNA interface (Figure 3c). This, therefore, entails that post-translational modification of H3R42 may alter histone–DNA (SHL-5 to SHL-7) contacts, propagating NCP unwrapping or breathing motion. This SHL-5 to SHL-7 region in the 3LZ0 system shows breathing motion after 6 μs, suggesting that kinetic communication among residues might play a part in the unwrapping (Figure S3). We note that this SHL-5 to SHL-7 region is shown to unwrap to a greater extent in these simulations, while we have observed using long-time simulations at higher salt concentrations^5^ that first the left side opens, followed by the right side, which is the side to unwrap via force as characterized by single-molecule FRET.^66^

The methylation of H3R42, a post-translational modification site at the DNA entry/exit region of the NCP, was seen to open the NCP by reducing the interaction between the double-stranded DNA and the histone core.^67^ Armeev and colleagues described H3R42 and adjacent amino acids (H3H39 and H3R49) as an H3 latch, revealing they were the first barrier to DNA unwrapping in the NCP, hence very important for gene regulation.^68^ Mechanistic models and cryo-EM suggest that remodelers drive nucleosomal motion via propagated DNA twist-defects, which intersect the latch/entry regions highlighted here.^69^ The residues H4R92 and H2BE76 are core oncogenic sites, and their mutation has been previously seen by Onyema et al. to destabilize the histone core through changes in hydrogen-bond dynamics.^43^ Figure 3d shows the conditional activity of H3R131, an oncogenic site with other amino acids in the histone core. The side-chain dynamics of H3R131 via long-range interactions exhibit both intra- and interhistone communications with other amino acid side chains in the NCP. H3R131 has been suggested to contribute to the stability of the tetramer–dimer structure of the nucleosome core particle.^70^ According to Ngubo and co-workers, H3R131 forms a hydrogen bond with H3Y99, H3D106, and H2AR99. Both intra- and interhistone interactions by H3R131 can promote nucleosome stability.

Mutations changed the dynamic memory of some amino acids in the nucleosome core particle. H2BE76K and H4R92T showed a shift in dynamical memory at the H4 and H2A α1 helices and the H4 loop 1 and α2 helices, all near the histone–DNA interface. In the H4R92T mutant system, H4T92 showed reduced dynamical memory, similar to that of the base pairs around the SHL-0 region. Both mutant systems showed shifts in H3R42, which is at the DNA entry/exit region, which has been suggested to affect DNA unwrapping (Figure 4). Our lab has previously shown that H2BE76K and H4R92T oncogenic mutations change the hydrogen-bond dynamics at the H4–H2B interface and also the free energy of binding at the H2A–H2B dimer–DNA interface.^43^

**Figure 4:**
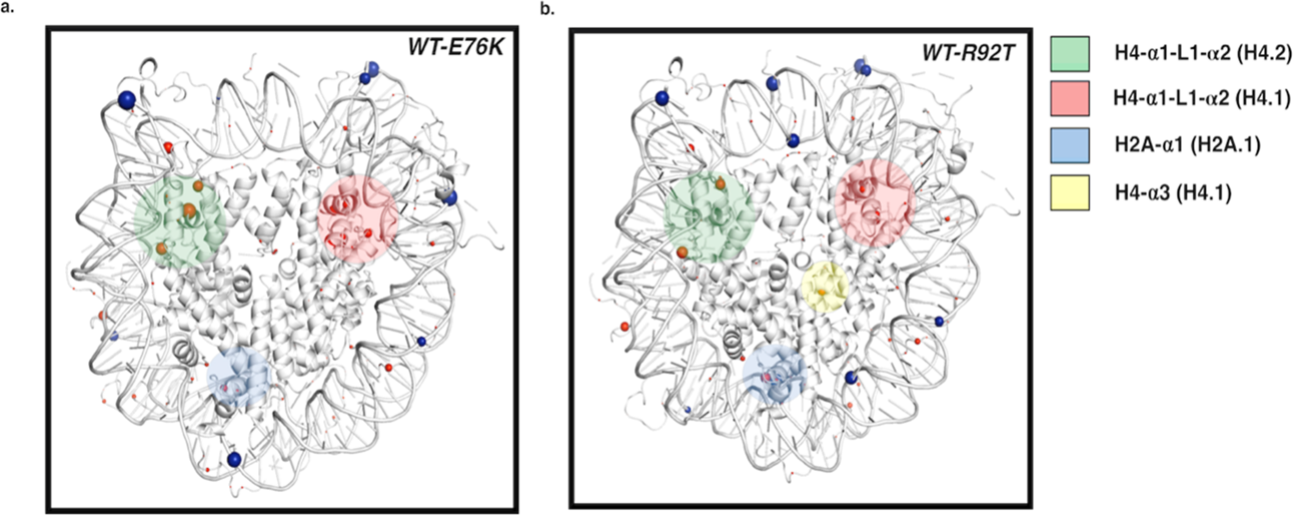
Difference in the dynamical memory between the WT and (a) H2BE76K and (b) H4R92T. There was a difference in the dynamical memory among residues in the H4 helices of α1, α2, α3, and loop 1, colored in green, red, and yellow. The α1 helix on histone H2A also had such a shift (blue). The sphere sizes are proportional to the differences in the dynamical memory between the wild type and each mutant system.

### 4.2 Principal Eigenvector Shows Communication between H2A–H2B Dimers

The nucleosome possesses dynamically connected domains, as indicated by its principal eigenvectors. The dynamically connected domains were nucleotide sequence-dependent, as shown in Figure 5a,b for the 1KX5 and 3LZ0 systems. The most dynamically connected domains are represented with red spheres, while the least are in green spheres. The domains with intermediate principal eigenvectors are in blue.

**Figure 5:**
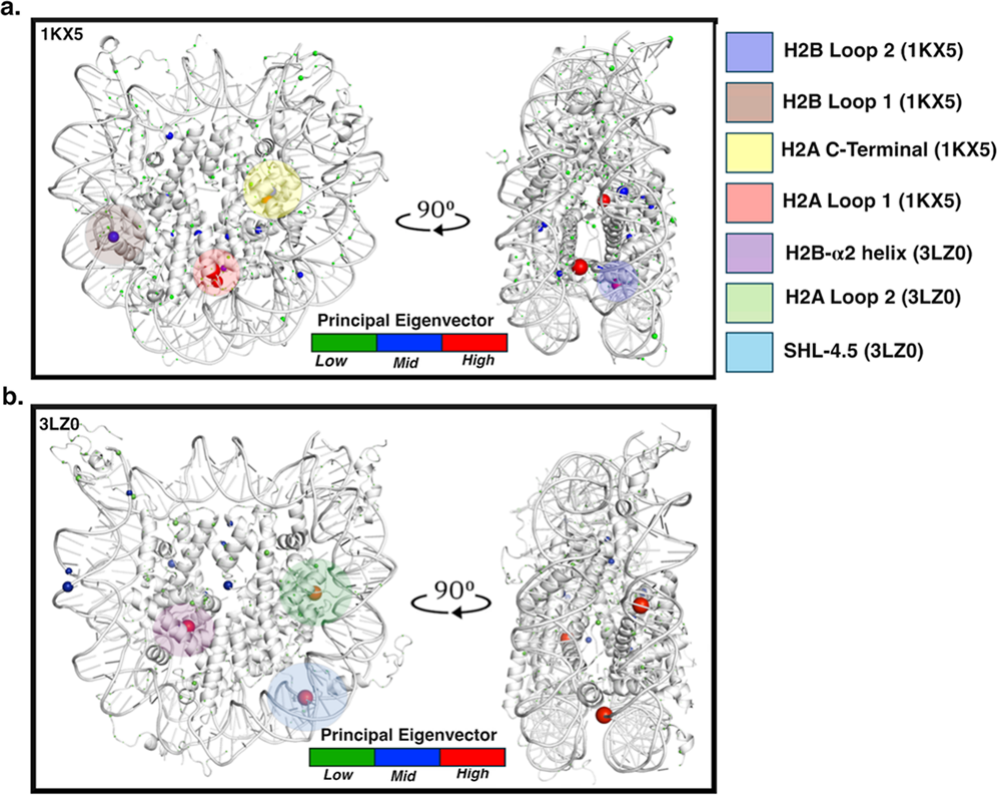
Most dynamically correlated regions of the (a) 1KX5 systems and the (b) 3LZ0 system shown at 0° and 90° rotations. The sphere size is proportional to the contribution of the residue to the principal eigenvector calculated from the symmetrized conditional activity matrix. In the 1KX5 system, regions whose degrees of freedom are kinetically connected include the C-terminal tail (yellow-shaded region) of H2A, loop 1 of H2A (red-shaded region), loop 1 (navy blue-shaded region) of H2B, and loop 2 (brown-shaded region) of H2B. In the 3LZ0 system, the α2 helix of H2B (violet-shaded region), loop 2 of H2A (green-shaded region), and the SHL-4.5 region (light-blue shaded region) of the Widom-601 DNA are kinetically correlated.

In the 1KX5 system, there are connections in the dihedral angle between histones H2A and H2B in the handshake region (Figure S4a,b) and between the two H2A–H2B dimers (Figures 5a and S4c). H2AY39 and H2BH49 primarily promote the kinetically connected domains in the first copy of the H2A–H2B dimer in loop 1 of each histone. In contrast, in the second copy of the H2A–H2B dimer, H2AI102 on the C-terminal tail, H2AQ84 on the α3 helix, and H2BT88 on loop 2 promote the kinetic connections of those domains. Similarly, H2AI102 and H2AY39 are suggested to foster dimer–dimer communication. Furthermore, both H2A and H2B dimers are kinetically connected to H4T71 in helix α2 and H4I46 in loop 1 (Figure S4d). In essence, there is a kinetic connection between the H3–H4 tetramer and both H2A and H2B dimers (Table S3).

Besides the kinetically connected domains above in both NCP systems, the 3LZ0 also shows a kinetic connection between loop 2 of H2A, promoted by H2AI79, the α2-helix of H2B, promoted by H2BF65, and G-45 in the SHL-4.5 region (Figures 5b and S5). Histone–DNA interaction at the SHL-4.5 region strains the base-sugar dihedral angle in G-45 (3^I^–5^I^ strand) from 290.5° to 331.5°, disturbing the Watson–Crick hydrogen bond (Figure S6). Remodeler complexes can actively distort nucleosomal DNA. For example, INO80 generates local DNA bulges during translocation, providing a structural route for the long-range kinetic couplings we observe across histone–DNA interfaces.^71^ There is also a tetramer–dimer kinetic connection at the C-terminus of both H3 (H3R134) and H4 (H4F100), loop 2 and the α1 helix of H4 (H4 V81 and H4I26), and the α3 helix on H3 (H3L126) in Figure S5.

### 4.3 Conditional Activity Shows Long-Range Correlated Dynamics

The conditional activity of residues in the nucleosome shows high sensitivity over long distances. The dynamic association between residues with respect to their separation in space is determined for the 1KX5, 3LZ0, H2BE76K, and H4R92T systems. The fraction of kinetically associated residues within a specific distance was the ratio of correlated residues with a conditional activity of at least 2 to all residues within that specific distance. The conditional activity cutoff of at least 2, corresponding to approximately 20% of the highest inter-residue conditional activity, was used. The dynamical memory, which is the conditional activity of a residue against itself (with a separation of 0 nm), showed that about 25% of the residues in the nucleosome showed strong kinetic association. As the spatial distance between residues increased, inter-residue association showed that the conditional activity was sensitive to residues up to 7.5 nm apart in the nucleosome system (Figure 6). At least 6% of residues that were 7.5 nm apart were seen to dynamically communicate. However, such communication was barely detectable between residues 8.0 nm apart.

**Figure 6:**
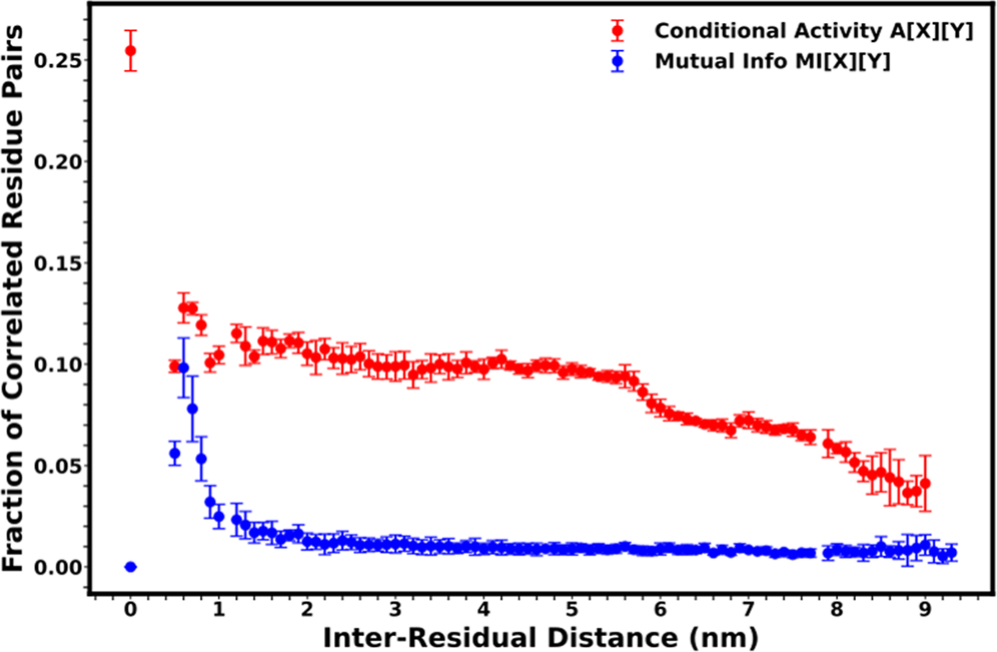
Comparison of the fraction of correlated dihedral angles using both conditional activity and mutual information against the distance between two residues in the nucleosome systems. At least 6% of correlated residues show conditional activity of their dihedral angles at a 7.5 nm separation for the nucleosome system. The conditional activity drastically reduces after 8.0 nm. Mutual information was sensitive to about 1.5 nm and was unnoticeable after 2.0 nm. The error bar is the standard error of the mean obtained from replicate nucleosome systems.

The fraction of correlated residue mutual information across all systems as a function of spatial distance was measured. The cross-residue mutual information magnitude decays quickly up to 1.5 nm and plateaus to zero above 2.0 nm (Figure 6). This shows that the conditional activity can detect fractions of correlated residue pairs over longer distances than that of mutual information.

## 5 Conclusion

Allosteric communication in biomolecular systems, particularly the nucleosome, plays a pivotal role in regulating cellular processes by coupling local perturbations to long-range conformational changes. Despite advancements in structural and thermodynamic analyses, understanding the kinetics of allosteric signaling, especially over extended spatial and temporal scales, remains a significant challenge. In this study, we introduce CONDACT (CONDitional ACTivity), a novel and open-source Python-based computational toolkit that quantifies kinetic correlations between molecular residues by evaluating time-resolved conditional activity. This approach leverages molecular dynamics (MD) simulations and builds upon Lin’s concept of conditional activity to map dynamically correlated domains and identify residues with high kinetic memory. Here, we apply CONDACT to four biological systems: two enzymes (lysozyme and PDZ3 domain of PSD-95) to validate the code and two nucleosome core particles (alpha-satellite DNA and Widom 601 sequence NCPs). Using dihedral angle transitions as kinetic clocks, we assess the dynamical memory and inter-residue conditional activity, revealing residue-specific and domain-level communication patterns, including known oncogenic mutation sites and post-translational modification (PTM) hotspots. Our analysis highlights kinetically significant domains such as the H4-α1 helix, acidic patch, and DNA SHL-4.5 regions. The SHL-4.5 region is the site for DNA repair or remodeling, where DNA polymerase β can bind, carrying out one-nucleotide excision repair within intact chromatin.^72^

These findings not only confirm previously characterized interactions but also uncover kinetically correlated residue pairs persisting at separations of up to 7 nm, underscoring the sensitivity and range of CONDACT. The principal eigenvectors of the symmetrized conditional activity matrix further elucidate domain-level allosteric coupling, providing mechanistic insights into nucleosome plasticity and regulation. Adhireksan and colleagues saw that RAPTA-T (a ruthenium compound) binding to the acidic patch of the NCP induces conformational changes that allosterically enhance the formation of auranofin adducts at distant 36 Å H3 sites (H113), highlighting a novel paradigm for epigenetic targeting through coordinated modulation of chromatin structure.^73^ Beyond local dynamics, heterochromatin proteins can reshape the nucleosome core and promote phase separation, offering an allosteric layer that meshes with our kinetic domain model.^74^ Genome-wide subnucleosomal mapping further indicates distinct nucleosome folding modes, consistent with multiple kinetic states captured by our domain-level eigenvectors.^75^ Overall, CONDACT bridges a critical gap in computational allostery by offering a kinetically centered perspective and a scalable analytical platform that can be applied across diverse biomolecular systems. As a freely accessible tool, it lays the groundwork for future studies aimed at detecting functional communication pathways, guiding drug discovery, and informing rational protein engineering design.^76^

As expected, CONDACT assigned high memory and inter-residue conditional activity, along with strong dynamical memory, to crucial domains corresponding to sites of post-translational modification (PTM) and oncogenic mutations, indicating strong dynamical coupling and functional significance. Key residues identified are within the acidic patch, such as H2AE56,^77^ or on the H3 tail within the latch region identified by Armeev et al.,^68^ such as H3R42. These sites are drug development hotspots, especially in the nucleosome, which is a challenging structure to target due to its tight packaging and dynamic regions like histone tails.^78–80^ Traditional drug design often struggles with the nucleosome because many meaningful interactions are transient or disordered.^79^ CONDACT overcomes this by focusing on how residues behave over time rather than relying only on entropic structural correlations. Residues with high conditional activity may act as communication hubs within the nucleosome and could be key to stabilizing the structure in the presence of disease-related mutations. The binding of transcription factors or chromatin remodelers can change these activity patterns, disrupting or enhancing communication across the nucleosome. Understanding how these shifts occur may provide insight into how mutations alter nucleosome function and suggest ways to correct these changes with drugs or other synthetically designed biomolecules. This approach offers a new path for targeting nucleosome-related diseases, such as cancer.

## Supplementary Material



## Data Availability

Analysis codes are available on GitHub at https://github.com/CUNY-CSI-Loverde-Laboratory/conditional_activity.git. Trajectories are available on Zenodo at https://zenodo.org/records/17193099.

## Notes

Table S1a and Table S1b: summary of simulation runs; Figure S1: dynamical memory of enzyme systems; Figure S2: conditional activity of enzymes and nucleosome systems; Figure S3: DNA backbone trace; Figure S4: principal eigenvectors and kinetically connected domains; Figure S5: kinetically connected domains; Figure S6: strain of the base-sugar dihedral angle in the 3LZO system; Table S2: inter-residue conditional activity; and Table S3: predicted kinetically connected domains (PDF)
