# Supplementary material for: Mapping Allosteric Communication in the Nucleosome with Conditional Activity
Source: J Chem Inf Model. 2026 Feb 24;66(5):2889–99. doi: 10.1021/acs.jcim.6c00317 (PMC12977051; doi:10.1021/acs.jcim.6c00317)
Supplement: Supplementary file 1 [file ci6c00317_si_001.pdf]

Supporting Information for

Mapping Allosteric Communication in the Nucleosome with Conditional Activity

Augustine C. Onyema[1,5], Chukwuebuka Dikeocha[2], Rutika Patel[1,5], Jonathan Moussa[3], and Sharon M. Loverde*[1,4,5]

[1] Department of Chemistry, College of Staten Island, The City University of New York, 2800 Victory Boulevard, Staten Island, New York 10314, United States

[2] Department of Computer Science, College of Staten Island, The City University of New York, 2800 Victory Boulevard, Staten Island, New York 10314, United States

[3] The Molecular Science Software Institute (MolSSI), Blacksburg, Virginia, USA.

[4] Ph.D. Program in Chemistry, Biochemistry, and Physics, The Graduate Center of the City University of New York, New York, New York 10016, United States

[5] Ph.D. Program in Biochemistry, The Graduate Center of the City University of New York, New York, New York 10016, United States

Corresponding Author: sharon.loverde@csi.cuny.edu

Supplementary Information Text

1. System Modelling/Simulation

1.1 System Modelling

Four systems were used for this study: Two enzyme systems and two NCPs. The enzyme systems were Lysozyme (1AKI.pbd) and PDZ3 (1BFE.pdb), while the NCPs were the alpha-satellite sequence (1KX5.pdb) and the Widom-601 sequence (3LZ0.pdb). AMBER force field was used to parameterize all the systems[1]. The amino acids of the protein in both enzymes and the nucleosome core particle (NCP) were parameterized with ff19SB[2], the DNA with OL15[3] , while OPC was used as the water model[4]. The Mn^{2+} in the crystal structure of the NCP was substituted with Mg^{2+}. Monovalent ions (Na^+ and Cl^-) in all systems were parameterized using the method of Joung and Cheetham,[5] while the method of Li and Merz was used for divalent ions (Mg^{2+} in the NCP)[6]. To properly model water-ion interactions, the Lennard-Jones parameters of monovalent and divalent ions were modified using the method of Kulkarni *et al*[7].

1.2 Simulation

Lysozyme and PDZ3

The enzyme systems were minimized using the steepest descent algorithm for 10 ps on GROMACS. Each system was equilibrated for 120 ps under the NVT ensemble and 1 ns under NPT ensemble[8, 9]. Each simulation was run for 3 µs, saving the trajectory once every 10 ps. The Nose-Hoover thermostat[10] was used to maintain the temperature of the system at 310 K while the pressure was kept at 1 bar using the Parrinello-Rahman barostat[11]. The SHAKE algorithm was applied to freeze hydrogen bonds for the simulation to be run with a 2 fs timestep[12]. Both the Particle Mesh Ewald (PME)[13] algorithm and van der Waals cut-off were kept at 10.0 Å to compute long-range electrostatic interaction and non-polar interaction, respectively.

Nucleosome Core Particle

For the NCP systems, minimization with both conjugate gradients and steepest descent algorithms was done for 15 ps with Amber18[14]. The system was equilibrated in NVT ensemble for 20 ps to 310 K[8, 9]. Furthermore, NPT equilibration at 1 bar using the Berendsen barostat[15] was done for 100 ns, maintaining the temperature of the system at 310 K with a Langevin thermostat. A timestep of 2 fs was used for this equilibration as the SHAKE algorithm[12] was implemented to freeze hydrogen atoms. The van der Waals cut off was 12 Å while the Particle Mesh Ewald (PME)[13] algorithm was used to calculate electrostatic interactions. The equilibrated system was run for 6 µs on Anton-2[16] as described in our previous work.

Conditional Activity Convergence

To assess convergence of the simulations and the robustness of the conditional activity analysis, each trajectory was divided into two halves, and the last 3 µs were analyzed and compared with the overall 6 µs. Similarity of conditional activity matrices (for 3 µs and 6 µs analyses) was evaluated using complementary metrics that probe both communication structure and strength[17]. The conditional activity matrices exhibited strong agreement across multiple complementary metrics, indicating stable communication patterns over time. Pearson correlation[18] of off-diagonal elements revealed a high degree of pattern similarity ($r = 0.91$), consistent with preservation of residue-to-residue communication, while cosine similarity (0.91) further confirmed alignment of the dominant signaling directions. Differences in coupling strength were modest, as reflected by a Frobenius norm[19] of 703 and a low mean squared error (0.43), indicating reweighting rather than reorganization of interactions. Network-level comparison after thresholding showed substantial overlap of communication pathways (Jaccard index ≈ 0.85)[20]. In addition, correlations of outgoing and incoming conditional activity strengths were high ($r = 0.93$ and $r = 0.94$, respectively), demonstrating that the same residues consistently act as dominant senders and receivers of dynamical information. Together, these results indicate that both the molecular dynamics trajectory and the conditional activity framework are well-converged, with stable allosteric communication emerging across independent simulation segments.

False Discovery Rate (FDR) Corrections

To control false positives arising from the large number of directed residue–residue interactions in the conditional activity (CA) network, we applied a false discovery rate (FDR) procedure to all tested CA edges[21]. For each directed residue pair (Y→X, represented as A[X][Y]), we computed an empirical one-sided p-value using a permutation-based null model. The number of permutations was selected as n_{perm} =1000 after testing multiple values and confirming stable p-value rankings and FDR outcomes. In this procedure, the transition-time series of the responding residue was randomized by circular time shifts over the full observation window, preserving each residue's intrinsic transition statistics while disrupting inter-residue temporal alignment. The observed CA value was then compared to the null distribution to quantify the probability of obtaining an equal or larger CA under the hypothesis of no directed coupling. P-values were subsequently corrected for multiple comparisons using the Benjamini–Hochberg FDR method with a target rate q=0.05. Edges with q-values ≤ 0.05 were considered statistically significant. This approach is essential for CA analyses because the number of candidate directed edges scales quadratically with system size, making nominal p-values prone to inflated discoveries. FDR correction ensures that the reported communication links represent a network with a controlled expected proportion of false positives.

Communication Route

For the conditional activity A[X][Y], the transition in Y is followed by a transition in X. To trace the communication route from one point to another, we use the conditional activity matrix and a matrix of the pairwise distance between residues in the system. The pairwise distance matrix was calculated with the center of geometry of each residue. Both the conditional activity matrix and the pairwise distance matrix are square (N x N). In tracing the communication path, residue positions are nodes, and the direction of conditional activity is an edge. We identify residues within a 10 Å cutoff that are kinetically correlated. For example, within a 10 Å for A[X][Y], Y is the source, and its transition is followed by a transition in X. That traces the first edge from Y to X. The X position becomes the New Y, and its conditional activity is used to find the new X within the next 10 Å in space. This tracing continues until the path of communication is traced from one point to another. Because a residue may be correlated with more than one other residue, we identified the top 10 residue networks for each source, thereby informing downstream network identification. This ensures a network of downstream correlated pairs can be formed. We also calculated the total score to rank the possibility of the communication path by summing all downstream conditional activities from the origin to the end.

$$Score = \sum_{1}^{n} A[X][Y]$$

Conditional Activity Difference Between WT and Mutant Systems

Evaluating if there is a shift in the conditional between the 1KX5 system and all mutants made from the 1KX5 wild-type system, we calculated the difference in conditional activity for the WT system and each mutant. The difference in the dynamical memory for each wild-type mutant pair was superimposed on the NCP structure as seen in Figure 4. The size of each sphere is proportional to the difference in dynamical memory between the systems.

$$\Delta A[X][Y]_{WT-mutant} = A[X][Y]_{WT} - A[X][Y]_{muant}$$

DNA Backbone Trace

The backbone trace of the DNA was done to capture breathing motion in both the 1KX5 and 3LZ0 wild-type systems after 6 µs. The systems were aligned to remove rotational and translational effects. The center of mass beteen phosphate atoms in the sugar-phosphate backbone for each base pair on the opposing strands was used to trace the position of the double-stranded DNA.

Mutual Information Analysis

The mutual information for each residue pair was calculated for each system using the dihedral-angle states. For amino acids, 0°≤ $\boldsymbol{\theta}$< 120° was assigned state X, 120°≤ $\boldsymbol{\theta}$< 240° was state Y, while 240°≤ $\boldsymbol{\theta}$< 360° was state Z. If an amino acid has a dihedral angle of 100°, 103°, and 210° at three different times, the states are X, X, and Y respectively. The mutual information of the states of two residues X and Y is defined by:

$$MI_{XY} = H_X + H_Y - H_{XY}$$

Dynamical Memory in Enzyme Systems

The dynamical memory identified amino acid residues in the active site of lysozyme and in both active and allosteric sites of synaptic protein PSD-95 (PDZ3), whose dynamics are statistically significant to the activity of the enzymes (**Figure S1a-b**). These amino acids in the lysozyme systems included Glu35, Asp52, Lys33, Ser36, and Asn59, which have been implicated to be either important for binding or catalysis in lysozyme[22]. This was the same with the PDZ3 systems in which Phe20, Asp43, and Tyr92 at the active and allosteric site had the highest dynamical memory, hence involved in the enzyme activity of PDZ3. The location of these amino acids is similar to helices, β-sheets, and loops, which have been proposed to be involved in binding or allosteric regulation[23]. The off-diagonal values reveal the inter-residue conditional activity and depict which direction of association between any two residues (**Figure S2a-b)**. A positive Tyr92-Pro3 and Phe20-Pro3 conditional activities (A[Y92][P3] = 9.336; A[F20][P3] = 8.263) suggest that the binding and allosteric sites of PDZ3 might be communicating with the N-terminal tail of the enzyme.

The principal eigenvector of the symmetrized conditional activity matrix for the PDZ3 system showed that the αA and αC helices and the tail of the βB strand are dynamically connected. Also, the binding site domains of lysozyme are dynamically connected (**Figure S1c-d**).

Table S1a: Enzyme all atom MD simulated systems

System	Lysozyme	PDZ3
**Position of Mutation**	None	None
**Residues mutated**	None	None
**Production run (Replica 1)**	3μs	3 μs

Table S1b: WT and mutants NCP all-atom MD simulated systems

System	WT (1KX5)	WT (3LZ0)	H4R92T	H2BE76K
**Position of Mutation**	None	None	α3 helix on H4	α2 helix on H2B
**Residues mutated**	None	None	Arg92 to Thr92	Glu76 to Lys76
**Production run (Replica 1)**	6 μs	6 μs	6 μs	6 μs
**Production run (Replica 2)**	6 μs	-	-	-

WT = Wild-Type
H4R92T = R92 to T92 on one copy of histone H4
H2BE76K = E76 to K76 on one copy of histone H2B

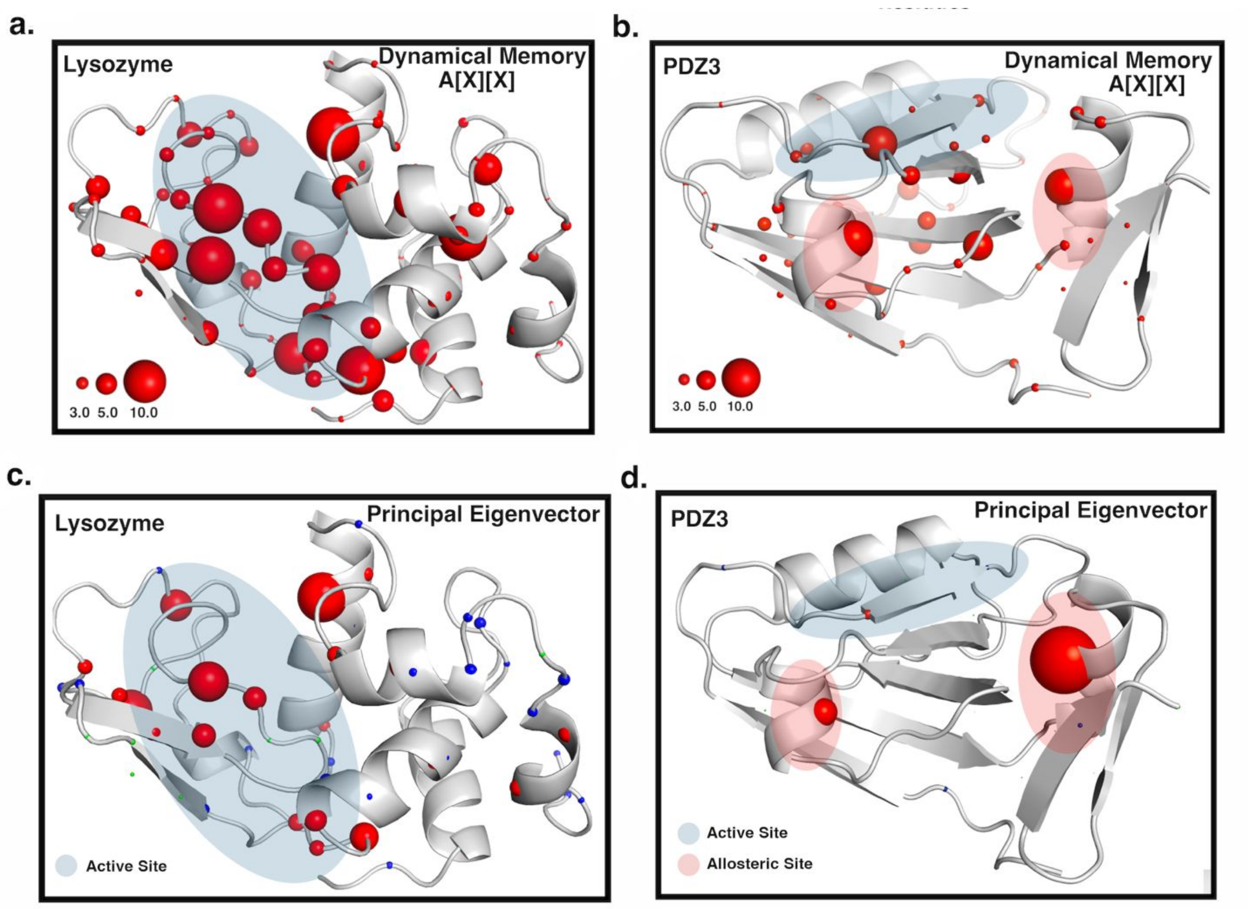

Suppl. Figure 1: Dynamical memory of all amino acids in (a) Lysozyme (b) PDZ3. The dynamical memory of the degree of freedom of amino acids at the active site (in blue) and allosteric site (in pink) is high; hence, their dynamics are statistically significant for enzyme activity. Principal eigenvector for (c) Lysozyme (d) PDZ3 reveals the regions with connected dynamics. The allosteric protein PDZ3 shows a sharp connection in dynamics among the allosteric αA and αC helices with the carboxylate binding loop, which interacts with ligands. Lysozyme shows a strong dynamic connection of helices and loops at the active site of the enzyme

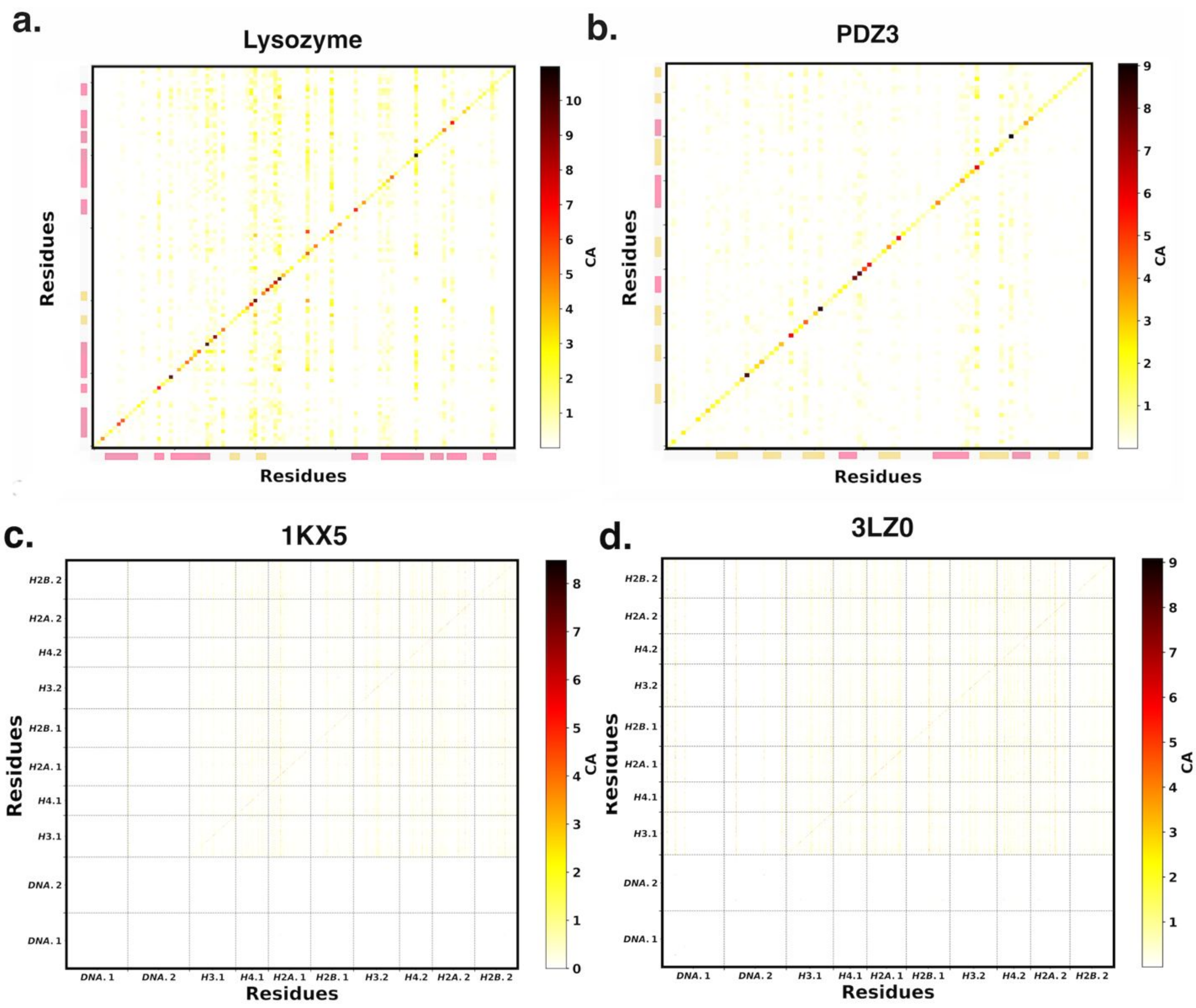

Suppl. Figure 2: Conditional activity of two enzymes; Lysozyme (1AKI.pdb) and Synaptic Protein PSD-95, PDZ3 (1BFE.pdb). Inter residue conditional activity heatmap for (a) Lysozyme (b) PDZ3. Pink rectangles depict α-helices, yellow rectangle **β**-sheets while interconnecting spaces are coils. Inter residue conditional activity heatmap for (c) 1KX5 (d) 3LZ0. High inter-residue conditional activity seen in the histone core while the double stranded DNA has little of no dynamics after 6 μs. High conditional activity in the histone core underscores amino acid communication is important for nucleosome dynamics.

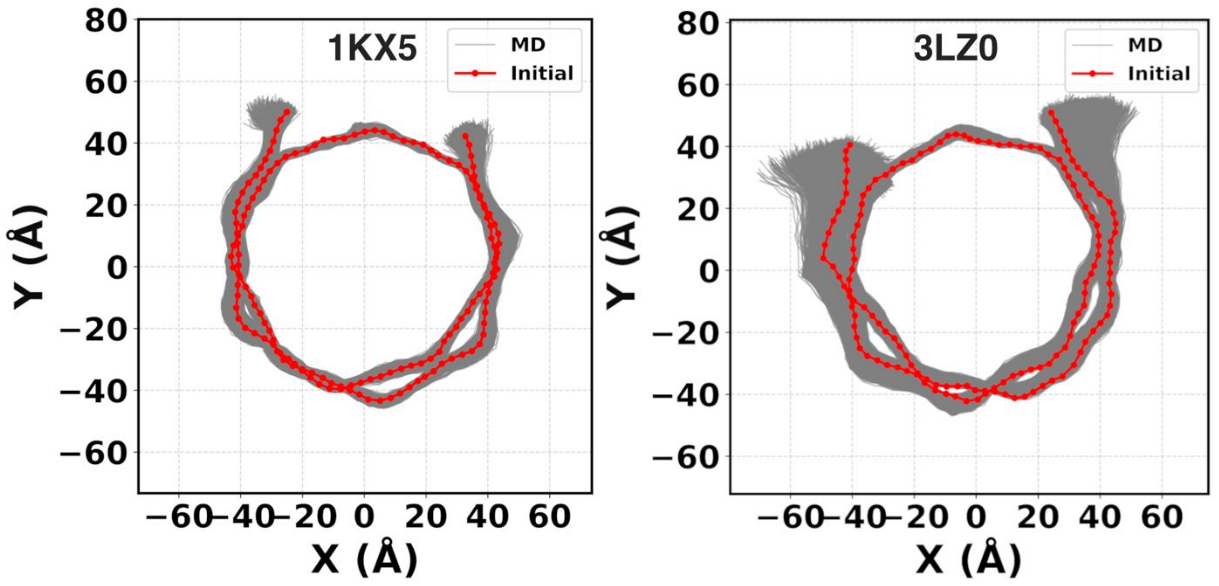

Suppl. Figure 3: DNA backbone trace for the 1KX5 and 3LZ0 systems after 6 μs. The simulation showed asymmetric unwrapping during breathing motion in 3LZ0. Breathing motion was observed in the SHL-5 to SHL-7 region. This region corresponds to the histone-DNA interface, where H3R42 and other residues exhibit high inter-residue conditional activity. The reference structure is in red while the simulated structure is in grey.

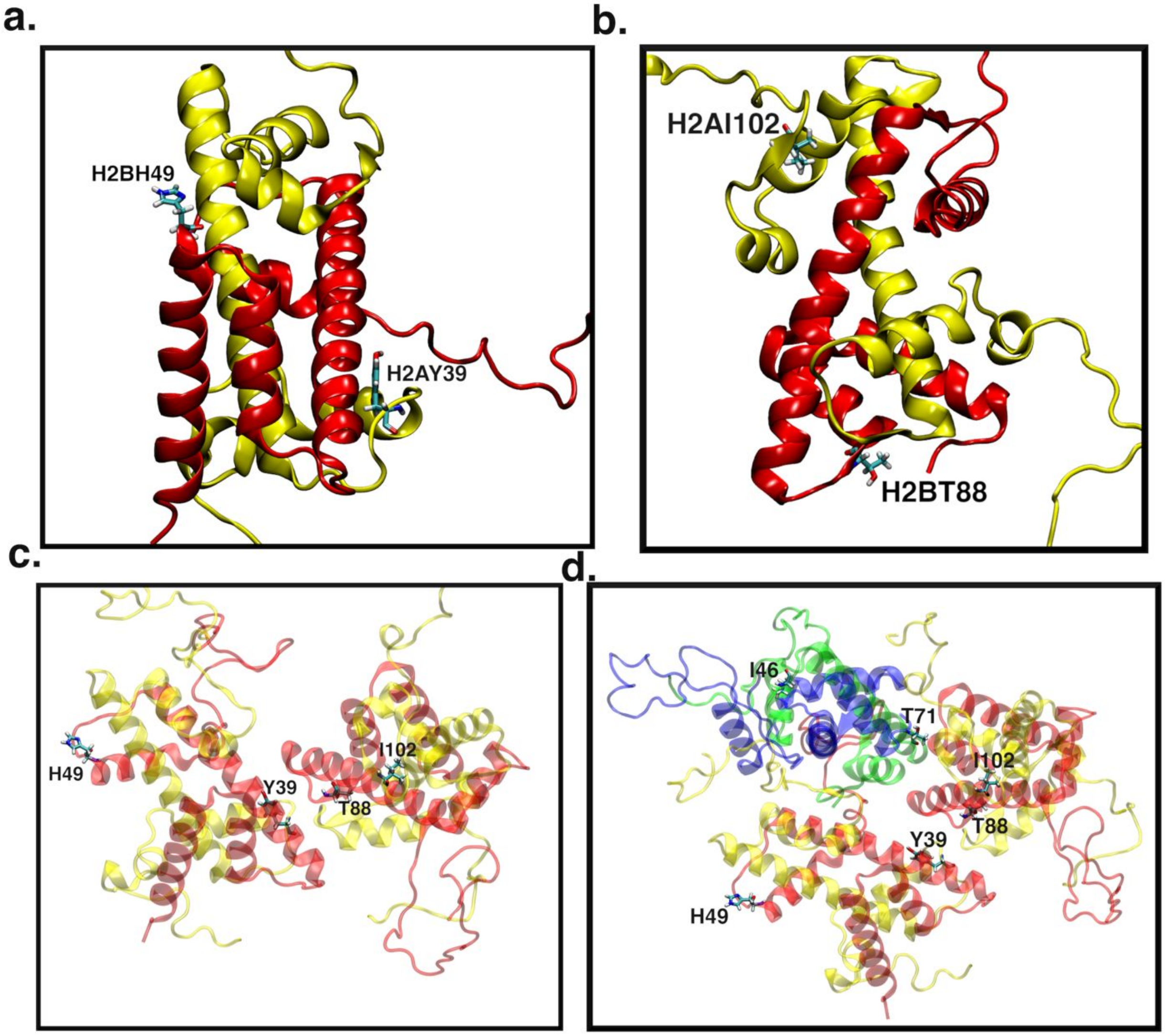

Suppl. Figure 4: Principal eigenvector showing kinetically connected domains in the nucleosome reveal major contributors to domain communication at (a) The first copy H2A-H2B dimer (b) The second copy H2A-H2B dimer (c) H2A-H2B dimers interface (d) The H2A-H2B dimers tetramer (H3-H4) interface. H2BH49, H2BT88, H2AY39, H2AI102, H4T71 and H4I46 were important for dimer-dimer and dimer-tetramer communication in both the wild-type 1KX5 and 3LZ0 systems.

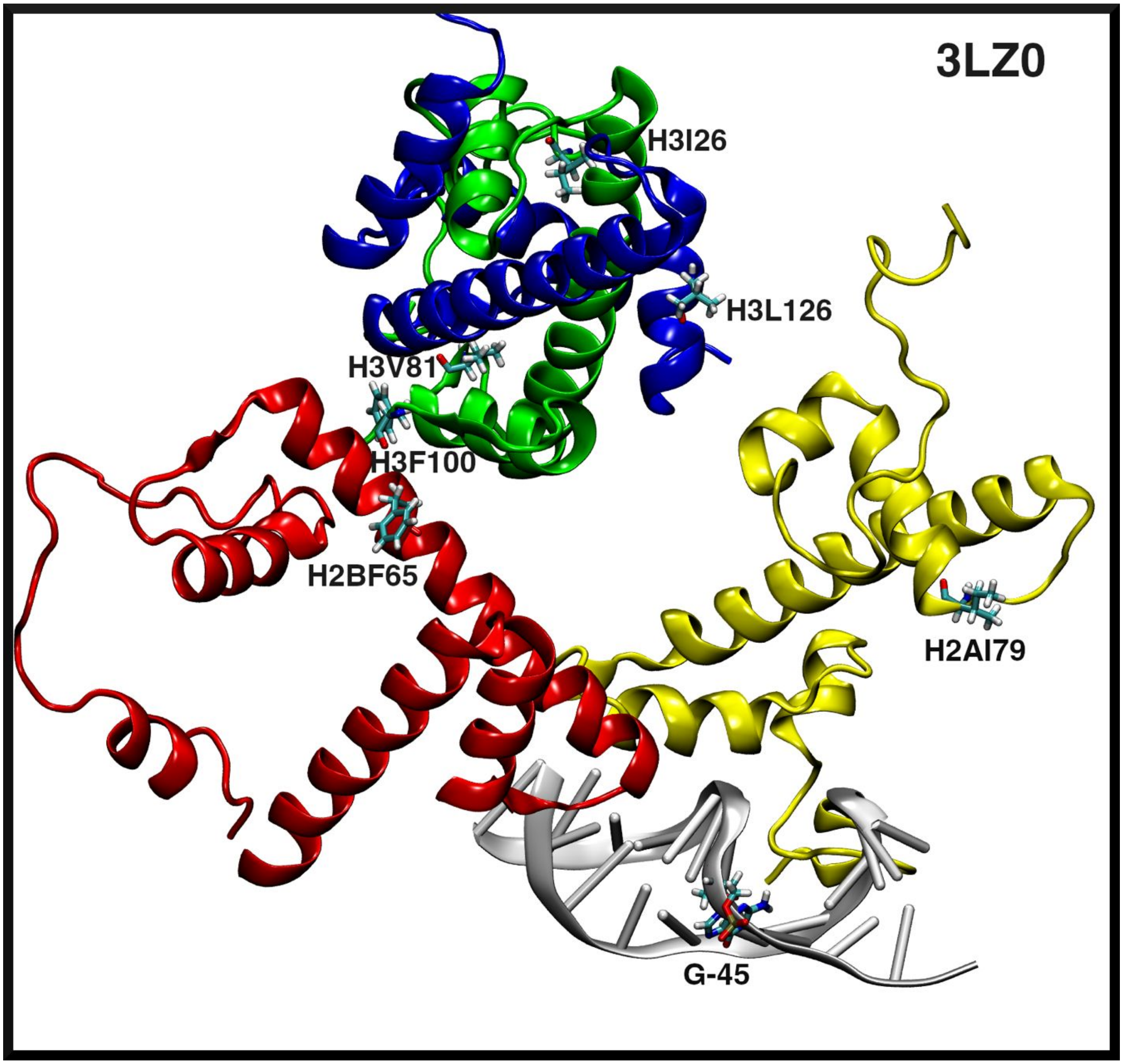

Suppl. Figure 5: Kinetically connected domains in the 3LZ0 systems among H2A-α3 helix (yellow), H2B-α2 helix (red), the SHL-4.5 region (grey). Tetramer-dimer kinetic connection was also seen at the α3 helix and C-terminus of H3 (blue) and loop 2 and α1 helix of H4 (green).

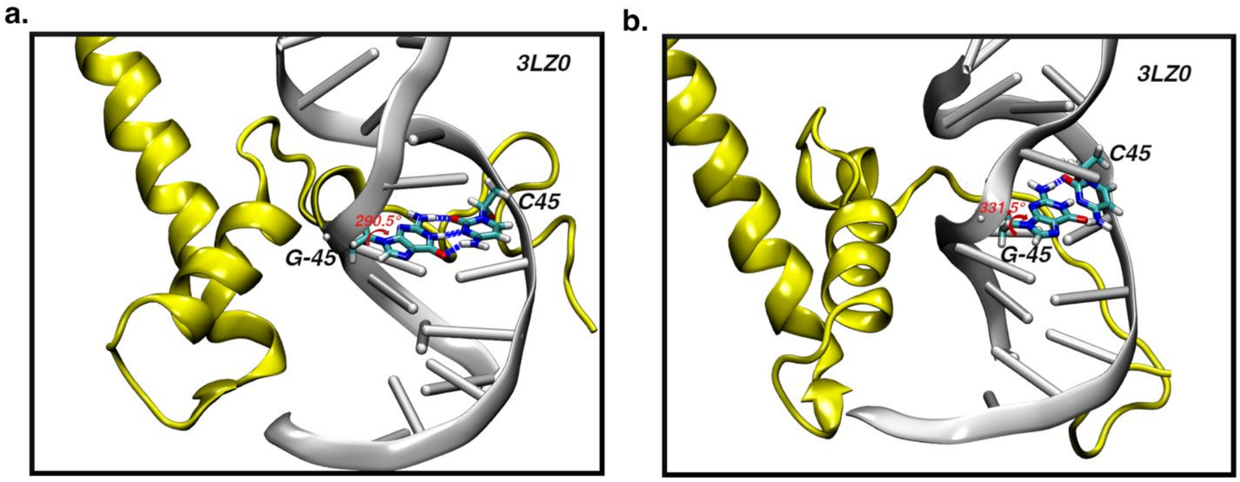

Suppl. Figure 6: Interaction between H2A.2 (yellow) and the SHL-4.5 region (grey) in the 3LZ0 systems strains the sugar-base dihedral angle in G-45 (3^{I}-5^{I} strand) from (a) 290.5° to (b) 331.5°. This strain affected the Watson-Crick base pairing between G-45 (3^{I}-5^{I} strand) and C45 (5^{I}-3^{I} strand).

Table S2: Inter-residue Conditional Activity in the 1KX5 System

Residue_X	Mut_Site_X	PTM_Site_X	Residue_Y	Mut_Site_Y	PTM_Site_Y	A[X][Y]	p_value	q_value	Sig_FDR
H3-T3	NO	NO	H2B-F70	YES	NO	2.51200606	0.0009995	0.01488041	TRUE
H3-K36	YES	YES	H2A-I102	NO	NO	3.31643701	0.00149925	0.01957628	TRUE
H3-E105	YES	NO	H2A-I102	NO	NO	3.04156299	0.00349825	0.03357056	TRUE
H3-K115	NO	YES	H4-T71	NO	NO	2.47596758	0.0049975	0.0419001	TRUE
H3-R131	YES	NO	H2A-I102	NO	NO	3.33454132	0.0049975	0.0419001	TRUE
H4-R3	YES	NO	H4-V81	NO	NO	2.20941899	0.00049975	0.0112127	TRUE
H4-R3	YES	NO	H2A-I102	NO	NO	2.96202931	0.00149925	0.01957628	TRUE
H4-R17	YES	NO	DNA-DA1	NO	NO	2.21973106	0.00149925	0.01957628	TRUE
H4-R17	YES	NO	H4-V81	NO	NO	2.37309375	0.0029985	0.03081685	TRUE
H4-R17	YES	NO	H2A-H31	YES	NO	2.63928355	0.00349825	0.03357056	TRUE
H4-R17	YES	NO	H2A-I102	NO	NO	4.72571205	0.00049975	0.0112127	TRUE
H4-E52	YES	NO	H2A-I102	NO	NO	4.13100503	0.00049975	0.0112127	TRUE
H2A-R11	YES	NO	H4-V81	NO	NO	2.12320864	0.00049975	0.0112127	TRUE
H2A-R11	YES	NO	H2A-I102	NO	NO	4.88314701	0.0009995	0.01488041	TRUE
H2A-L85	NO	NO	H3-R131	YES	NO	2.05676407	0.0009995	0.01488041	TRUE
H2A-E92	YES	NO	H2A-H31	YES	NO	2.2767494	0.00549725	0.04445239	TRUE
H2A-E92	YES	NO	H2A-I102	NO	NO	5.12815188	0.00049975	0.0112127	TRUE
H2B-K24	NO	NO	H2A-R11	YES	NO	2.92785437	0.0009995	0.01488041	TRUE
H2B-E35	YES	NO	H4-V81	NO	NO	2.1972891	0.00049975	0.0112127	TRUE
H2B-E35	YES	NO	H2A-Y39	NO	NO	2.11230238	0.0009995	0.01488041	TRUE
H2B-E35	YES	NO	H2A-I102	NO	NO	3.11226578	0.00049975	0.0112127	TRUE
H2B-D51	YES	NO	H4-I46	NO	NO	3.09518171	0.005997	0.04615084	TRUE
H2B-E71	YES	NO	H2A-I102	NO	NO	3.3182794	0.00149925	0.01957628	TRUE
H2B-N84	NO	NO	H2A-R11	YES	NO	2.68986768	0.00049975	0.0112127	TRUE

H2B-E113	YES	NO	H3-V71	NO	NO	2.3808597	0.001999	0.02341476	TRUE
H2B-K120	NO	YES	H4-R36	NO	NO	2.12386703	0.001999	0.02341476	TRUE
H3-R2	YES	YES	H4-I46	NO	NO	3.43389645	0.001999	0.02341476	TRUE
H3-R2	YES	YES	H2A-I102	NO	NO	4.12692291	0.00049975	0.0112127	TRUE
H3-S10	NO	NO	H2B-F70	YES	NO	2.34991498	0.003998	0.03618645	TRUE
H3-K14	NO	YES	DNA-DA1	NO	NO	2.27704725	0.00449775	0.03915255	TRUE
H3-K14	NO	YES	H2A-I102	NO	NO	3.56244395	0.00249875	0.02745179	TRUE
H3-K14	NO	YES	H2B-V111	NO	NO	2.17768867	0.00349825	0.03357056	TRUE
H3-L65	NO	NO	H3-R42	NO	YES	5.39281695	0.001999	0.02341476	TRUE
H3-D81	YES	NO	DNA-DA1	NO	NO	2.47666788	0.0009995	0.01488041	TRUE
H3-D81	YES	NO	H2A-I102	NO	NO	3.61409589	0.00049975	0.0112127	TRUE
H4-S1	YES	NO	H2A-I102	NO	NO	2.54573177	0.003998	0.03618645	TRUE
H4-R3	YES	NO	H2A-Y39	NO	NO	2.50368889	0.0029985	0.03081685	TRUE
H4-R3	YES	NO	H2A-I102	NO	NO	3.99748201	0.0009995	0.01488041	TRUE
H4-R45	YES	NO	H4-V81	NO	NO	2.05427329	0.0009995	0.01488041	TRUE
H4-R45	YES	NO	H2A-I102	NO	NO	5.58791706	0.0009995	0.01488041	TRUE
H4-E52	YES	NO	H2A-I102	NO	NO	3.70428036	0.00049975	0.0112127	TRUE
H4-K77	NO	YES	H2A-I102	NO	NO	2.56030189	0.00349825	0.03357056	TRUE
H4-K91	NO	YES	H2A-L23	NO	NO	2.36580535	0.00549725	0.04445239	TRUE
H2A-R11	YES	NO	H2A-I102	NO	NO	6.98960123	0.00049975	0.0112127	TRUE
H2A-R17	YES	NO	H3-V71	NO	NO	2.50315905	0.00549725	0.04445239	TRUE
H2A-L83	NO	NO	H2A-H31	YES	NO	2.38595358	0.0009995	0.01488041	TRUE
H2B-K11	NO	NO	H2A-R11	YES	NO	3.14997426	0.00049975	0.0112127	TRUE
H2B-H49	NO	NO	H2A-H31	YES	NO	2.53977714	0.00349825	0.03357056	TRUE
H2B-L102	NO	NO	H2A-H31	YES	NO	2.86011163	0.00249875	0.02745179	TRUE
H2B-E113	YES	NO	H4-T71	NO	NO	2.61269975	0.0029985	0.03081685	TRUE

Table S3: Predicted Kinetically Connected domains with Residues contributing to Principal Eigenvector and Dynamical Memory in the NCP Systems

Histone	Domain	Contributors to eigenvector	High Dynamical memory
**H3**	αN helix	Tyr 54	—
**H3**	αN–α1 loop/ α1 helix	Leu 61; Gln 76	Arg 63
**H3**	α2 helix	Phe 104	—
**H3**	α3 helix	Leu 126; Ile 130; Arg 129; Arg 134	—
**H4**	α1 helix	Ile 26; Ile 29; Thr 30; Arg 39	Asp 24, Arg 35, Arg 36, Arg 40
**H4**	Loop 1	Ile 46;	Lys 44
**H4**	α2 helix	Val 70; Thr 71; Tyr 72	Glu 53, Arg 55
**H4**	Loop 2	Val 81	—
**H4**	α3 helix	Phe 100	Arg 92
**H2A**	Loop 1	Tyr 39	—
**H2A**	α3 helix	Ile 78; Gln 84	—
**H2A**	C-terminal tail/ docking domain	Glu 92; Ile 102	—
**H2B**	Loop 1	His 49; Ile 54	—
**H2B**	α2 helix	Phe 65; Phe 70	Phe 70
**H2B**	Loop 2	Tyr 83; Thr 88	—
**H2B**	α3 helix	Ile 89; Leu 100	—
**H2B**	αC-terminal tail	Thr 115	—

References

(1) Ponder, J. W.; Case, D. A. Force fields for protein simulations. In *Protein Simulations*, Daggett, V. Ed.; Advances in Protein Chemistry, Vol. 66; 2003; pp 27-+.

(2) Tian, C.; Kasavajhala, K.; Belfon, K. A. A.; Raguette, L.; Huang, H.; Migues, A. N.; Bickel, J.; Wang, Y. Z.; Pincay, J.; Wu, Q.; et al. ff19SB: Amino-Acid-Specific Protein Backbone Parameters Trained against Quantum Mechanics Energy Surfaces in Solution. *Journal of Chemical Theory and Computation* **2020**, *16* (1), 528-552. DOI: 10.1021/acs.jctc.9b00591.

(3) Zgarbová, M.; Sponer, J.; Otyepka, M.; Cheatham, T. E.; Galindo-Murillo, R.; Jurecka, P. Refinement of the Sugar-Phosphate Backbone Torsion Beta for AMBER Force Fields Improves the Description of Z- and B-DNA. *Journal of Chemical Theory and Computation* **2015**, *11* (12), 5723-5736. DOI: 10.1021/acs.jctc.5b00716.

(4) Izadi, S.; Anandakrishnan, R.; Onufriev, A. V. Building Water Models: A Different Approach. *Journal of Physical Chemistry Letters* **2014**, *5* (21), 3863-3871. DOI: 10.1021/jz501780a.

(5) Joung, I. S.; Cheatham, T. E. Determination of alkali and halide monovalent ion parameters for use in explicitly solvated biomolecular simulations. *Journal of Physical Chemistry B* **2008**, *112* (30), 9020-9041. DOI: 10.1021/jp8001614.

(6) Li, Z.; Song, L. F.; Li, P. F.; Merz, K. M. Systematic Parametrization of Divalent Metal Ions for the OPC3, OPC, TIP3P-FB, and TIP4P-FB Water Models. *Journal of Chemical Theory and Computation* **2020**, *16* (7), 4429-4442. DOI: 10.1021/acs.jctc.0c00194.

(7) Kulkarni, M.; Yang, C.; Pak, Y. Refined Alkali Metal Ion Parameters for the OPC Water Model. *Bulletin of the Korean Chemical Society* **2018**, *39* (8), 931-935. DOI: 10.1002/bkcs.11527.
(8) Li, D. Z.; Chen, Z. F.; Zhang, Z. J.; Liu, J. Understanding Molecular Dynamics with Stochastic Processes via Real or Virtual Dynamics. *Chinese Journal of Chemical Physics* **2017**, *30* (6), 735-760. DOI: 10.1063/1674-0068/30/cjcp1711223.
(9) Zhang, Z.; Liu, X.; Yan, K.; Tuckerman, M. E.; Liu, J. Unified Efficient Thermostat Scheme for the Canonical Ensemble with Holonomic or Isokinetic Constraints via Molecular Dynamics. *The Journal of Physical Chemistry A* **2019**, *123* (28), 6056-6079. DOI: 10.1021/acs.jpca.9b02771.
(10) Evans, D. J.; Holian, B. L. The Nose–Hoover thermostat. *The Journal of Chemical Physics* **1985**, *83* (8), 4069-4074. DOI: 10.1063/1.449071.
(11) Parrinello, M.; Rahman, A. Polymorphic transitions in single crystals: A new molecular dynamics method. *Journal of Applied Physics* **1981**, *52* (12), 7182-7190. DOI: 10.1063/1.328693.
(12) Ryckaert, J.-P.; Ciccotti, G.; Berendsen, H. J. C. Numerical integration of the cartesian equations of motion of a system with constraints: molecular dynamics of n-alkanes. *Journal of Computational Physics* **1977**, *23* (3), 327-341. DOI: 10.1016/0021-9991(77)90098-5.
(13) Darden, T.; York, D.; Pedersen, L. Particle mesh Ewald: An <i>N</i>·log(<i>N</i>) method for Ewald sums in large systems. *The Journal of Chemical Physics* **1993**, *98* (12), 10089-10092. DOI: 10.1063/1.464397.
(14) Case, D. A.; Cheatham, T. E.; Darden, T.; Gohlke, H.; Luo, R.; Merz, K. M.; Onufriev, A.; Simmerling, C.; Wang, B.; Woods, R. J. The Amber biomolecular simulation programs. *Journal of Computational Chemistry* **2005**, *26* (16), 1668-1688. DOI: 10.1002/jcc.20290.
(15) Berendsen, H. J. C.; Postma, J. P. M.; Van Gunsteren, W. F.; Dinola, A.; Haak, J. R. Molecular dynamics with coupling to an external bath. *The Journal of Chemical Physics* **1984**, *81* (8), 3684-3690. DOI: 10.1063/1.448118.
(16) Shaw, D. E.; Grossman, J. P.; Bank, J. A.; Batson, B.; Butts, J. A.; Chao, J. C.; Deneroff, M. M.; Dror, R. O.; Even, A.; Fenton, C. H.; et al. Anton 2: Raising the Bar for Performance and Programmability in a Special-Purpose Molecular Dynamics Supercomputer. 2014, IEEE. DOI: 10.1109/sc.2014.9.
(17) Ichiye, T.; Karplus, M. Collective motions in proteins: A covariance analysis of atomic fluctuations in molecular dynamics and normal mode simulations. *Proteins: Structure, Function, and Bioinformatics* **1991**, *11* (3), 205-217. DOI: 10.1002/prot.340110305.
(18) Pearson, K. VII. Note on regression and inheritance in the case of two parents. *Proceedings of the Royal Society of London* **1895**, *58* (347-352), 240-242. DOI: 10.1098/rspl.1895.0041.
(19) Eckart, C.; Young, G. The Approximation of One Matrix by Another of Lower Rank. *Psychometrika* **1936**, *1* (3), 211-218. DOI: 10.1007/bf02288367.
(20) Jaccard, P. Distribution de la flore alpine dans le bassin des Dranses et dans quelques régions voisines. *Bulletin de la Société Vaudoise des Sciences Naturelles* **1901**, *37*, 241-272. DOI: 10.5169/seals-266440.
(21) Benjamini, Y.; Hochberg, Y. Controlling the False Discovery Rate: A Practical and Powerful Approach to Multiple Testing. *Journal of the Royal Statistical Society Series B: Statistical Methodology* **1995**, *57* (1), 289-300. DOI: 10.1111/j.2517-6161.1995.tb02031.x.

(22) Held, J.; Van Smaalen, S. The active site of hen egg-white lysozyme: flexibility and chemical bonding. *Acta Crystallographica Section D Biological Crystallography* **2014**, *70* (4), 1136-1146. DOI: 10.1107/s1399004714001928.
(23) Stevens, A. O.; He, Y. Allosterism in the PDZ Family. *International Journal of Molecular Sciences* **2022**, *23* (3), 1454. DOI: 10.3390/ijms23031454.
